# Filamin B restricts vaccinia virus spread and is targeted by vaccinia virus protein C4

**DOI:** 10.1128/jvi.01485-23

**Published:** 2024-02-27

**Authors:** Iliana Georgana, Simon R. Scutts, Chen Gao, Yongxu Lu, Alice A. Torres, Hongwei Ren, Edward Emmott, Jinghao Men, Keefe Oei, Geoffrey L. Smith

**Affiliations:** 1 Department of Pathology, University of Cambridge, Cambridge, United Kingdom; 2 Sir William Dunn School of Pathology, University of Oxford, Oxford, United Kingdom; Northwestern University Feinberg School of Medicine, Chicago, Illinois, USA

**Keywords:** vaccinia virus, protein C4, NF-κB, DNA sensing, filamin B, cytoskeleton, virus virulence, virus restriction factor, virus-host interactions

## Abstract

Vaccinia virus (VACV) is a large DNA virus that encodes scores of proteins that modulate the host immune response. VACV protein C4 is one such immunomodulator known to inhibit the activation of both the NF-κB signaling cascade and the DNA-PK-mediated DNA sensing pathway. Here, we show that the N-terminal region of C4, which neither inhibits NF-κB nor mediates interaction with DNA-PK, still contributes to virus virulence. Furthermore, this domain interacts directly and with high affinity to the C-terminal domain of filamin B (FLNB). FLNB is a large actin-binding protein that stabilizes the F-actin network and is implicated in other cellular processes. Deletion of FLNB from cells results in larger VACV plaques and increased infectious viral yield, indicating that FLNB restricts VACV spread. These data demonstrate that C4 has a new function that contributes to virulence and engages the cytoskeleton. Furthermore, we show that the cytoskeleton performs further previously uncharacterized functions during VACV infection.

**IMPORTANCE:**

Vaccinia virus (VACV), the vaccine against smallpox and monkeypox, encodes many proteins to counteract the host immune response. Investigating these proteins provides insights into viral immune evasion mechanisms and thereby indicates how to engineer safer and more immunogenic VACV-based vaccines. Here, we report that the N-terminal domain of VACV protein C4 interacts directly with the cytoskeletal protein filamin B (FLNB), and this domain of C4 contributes to virus virulence. Furthermore, VACV replicates and spreads better in cells lacking FLNB, thus demonstrating that FLNB has antiviral activity. VACV utilizes the cytoskeleton for movement within and between cells; however, previous studies show no involvement of C4 in VACV replication or spread. Thus, C4 associates with FLNB for a different reason, suggesting that the cytoskeleton has further uncharacterized roles during virus infection.

## INTRODUCTION

Poxviruses are large DNA viruses that infect either insects or chordates (1). Variola virus is the most infamous poxvirus and the causative agent of smallpox, an extinct human disease that was eradicated by vaccination with vaccinia virus (VACV) (2). Following the eradication of smallpox, interest in VACV has endured because the virus genome can be engineered to express foreign genes (3, 4) and so create candidate vaccines for other infectious diseases (5–7). VACV is also being developed as an oncolytic agent (8, 9) and is a good model for studying virus-host interactions. VACV has a dsDNA genome of about 190 kbp and encodes approximately 200 proteins (10), of which one-third to one-half counteract the host immune response to infection (11–14). This paper concerns one such immunomodulatory protein called C4.

VACV strain Western Reserve (WR) protein C4 is a 37-kDa protein that is expressed early during infection and is present in both the nucleus and cytoplasm (15). A mutant virus lacking gene *C4L*, called v∆C4, replicated and spread normally in cultured cells but had reduced virus virulence and induced an altered immune response to infection (15, 16). Functionally, C4 inhibits the activation of the NF-κB signaling cascade (15) and the DNA-PK-mediated DNA sensing pathway by binding to Ku heterodimers (16). C4 shares 44% amino acid (aa) identity with another VACV WR protein, called C16, which is also a non-essential, early protein that affects virulence and the host response to infection (17). C4 and C16 each have distinct N- and C-terminal domains, with the C-terminal domain being more closely related (56% aa identity) and sharing a common function, namely binding the Ku proteins (16, 18), which are part of the DNA-PK trimeric complex. The interaction with the Ku proteins prevents their binding to DNA, consequently inhibiting DNA-PK-mediated DNA sensing (18, 19). Recently, the cryoEM structure of C16 bound to Ku provided a molecular explanation of how C16 (and C4) blocks Ku-DNA interaction and thereby cytosolic DNA sensing (20). In addition, it was shown recently that the Ku proteins interact with cyclic GMP-AMP synthetase (cGAS) and promote DNA binding and condensation of cGAS, thereby enabling DNA sensing (21). The N-terminal domains of C4 and C16 are more divergent and perform distinct functions. C16, but not C4 (16), binds to prolyl hydroxylase domain-containing protein 2 (PHD2) and thereby induces a hypoxic response during normoxia (22) and reprograms central energy metabolism (23). To study C4 further, and particularly the function of the N-terminal domain, we performed an unbiased proteomic search for C4-binding proteins and identified filamin-B (FLNB) as an interacting partner.

The FLN family has three members: FLNA, B, and C. FLNs are large, actin-binding proteins that organize three-dimensional actin cytoskeletal networks and link them to cell membranes by binding to transmembrane proteins (24). FLNA is the most abundant and well-studied FLN, and FLNC is mainly expressed in muscle cells and to a low level in other tissues (25, 26). FLNB is expressed at the highest level in endothelial cells and widely in other cell types at lower levels (27). Human FLNB is a 2,602 aa protein with several domains. Its N-terminal region contains the actin-binding domain, which consists of two calponin-homology domains. These are followed by 24 repeated immunoglobulin-like domains that are interrupted by two hinge regions. Hinge 1 (H1) is located between repeats 15 and 16 (28), whereas hinge 2 (H2) lies between repeats 23 and 24 (29). Overall, FLNB exhibits about 70% aa identity with the other FLNs, with their hinge regions being the most divergent (30, 31). FLNB, like other FLNs, is found as either a homo- or heterodimer with other FLNs, with dimerization being mediated via repeat 24 near the C terminus. FLNB has important roles in cellular architecture and motility by stabilizing the cytoskeleton but also has important roles for receptor activation and signal transduction (32). For instance, it has a role in innate immune signaling by acting as a scaffold for mitogen-activated protein kinases (MAPK) in response to type I IFN sensing (33).

Here, we have identified a new function for VACV protein C4, namely its interaction with the cytoskeletal protein FLNB. This interaction is direct, occurs via the N-terminal region of C4 and the C-terminal region of FLNB, and does not require FLNB dimerization. The N-terminal domain of C4 is shown to contribute to virulence and a role for FLNB as an antiviral factor is demonstrated by observing an increased plaque size and infectious virus yield in cells lacking FLNB compared to controls. Overall, this study defines a new role for C4 and reveals a new function for the cytoskeleton during viral infection.

## RESULTS

### C4-N interacts with FLNs

**Fig 1:**
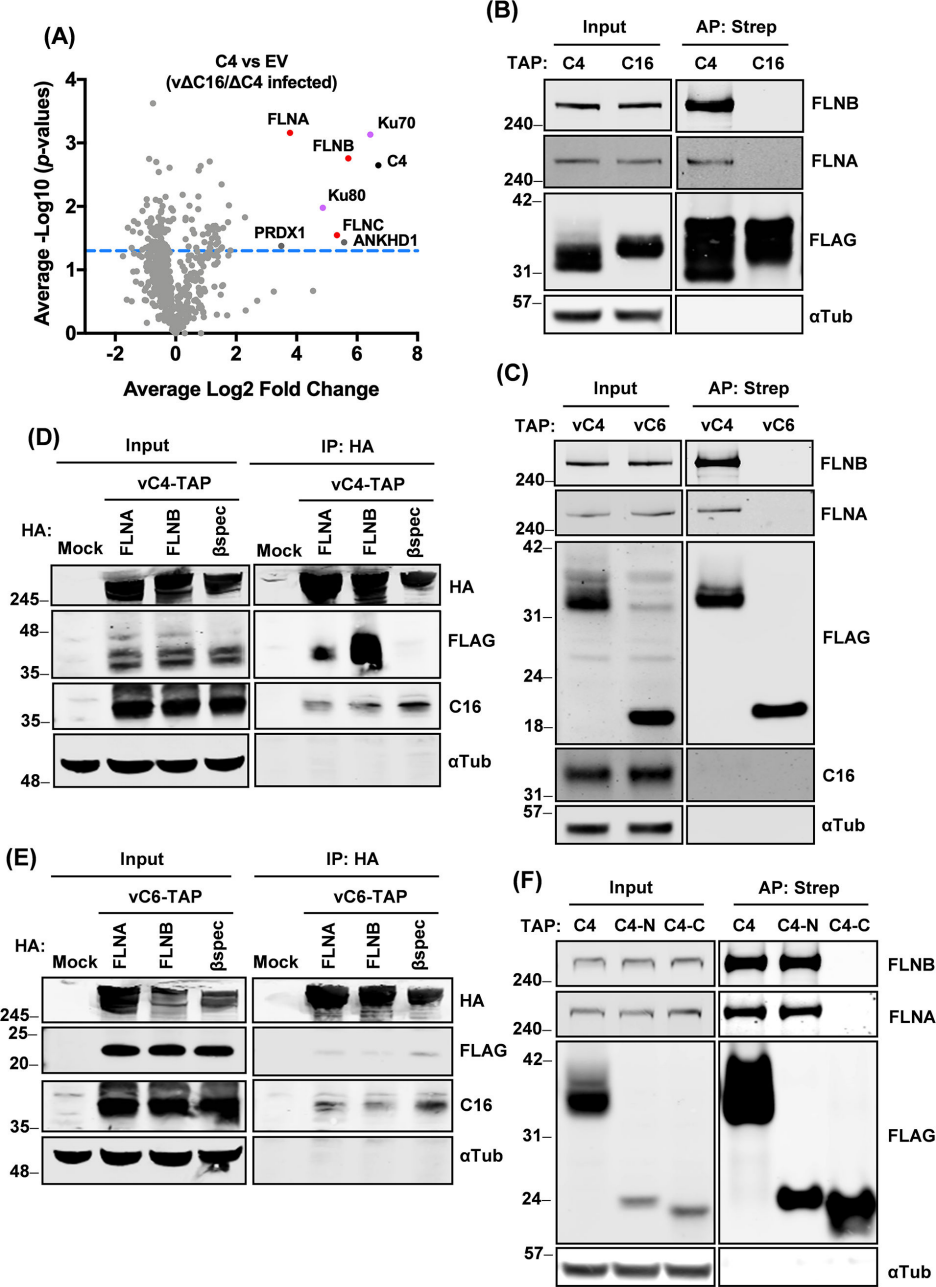
C4 interacts with FLNs via the C4 N-terminal domain. (**A**) HEK293T cells were transfected in triplicate with TAP-tagged C4 or EV for 16 h and then infected with vΔC16/ΔC4 at a multiplicity of infection (MOI) of 5 for 7 h. Cells were lysed and subjected to AP via the strep tag. The AP samples were then analyzed by TMT proteomics. Log2 ratios of the mean of three TAP-C4 pull-downs (*x*-axis) are plotted versus −Log10 of the *P*-values (*y*-axis) derived from a Student’s *t*-test. The threshold of *P*-value = 0.05 is indicated with the blue dashed line. C4 is shown with a black dot, FLNA/B/C are indicated in red dots, and the known C4-interacting partners Ku70/80 are shown on purple dots. Low significance hits PRDX1 and ANKHD1 are indicated as gray dots. (**B**) HEK293T cells were transfected for 24 h with plasmids encoding TAP-tagged C4 or C16. Cells were lysed and tagged proteins were purified as above, separated by NuPAGE and analyzed by immunoblotting with the indicated antibodies. (**C**) HeLa cells were infected with vC4-TAP or vC6-TAP at an MOI of 10 for 6 h. Cells were then lysed and subjected to AP. Proteins were separated by NuPAGE and analyzed by immunoblotting. (**D and E**) HEK293T cells were transfected for 24 h with plasmids encoding HA-tagged FLNA, FLNB, or β-spectrin. Then, the cells were infected with (**D**) vC4-TAP or (**E**) vC6-TAP at an MOI of 2 for 16 h. Cells were then lysed, and HA-tagged proteins were immunoprecipitated (IP) and proteins were immunoblotted with the indicated antibodies. (**F**) HEK293T cells were transfected for 24 h with plasmids encoding TAP-tagged C4, C4 residues 1–156 (**C4-N**) or C4 residues 157–316 (**C4-C**). Cells were lysed and tagged proteins were purified and analyzed as in panel **B**. Data shown are representative of three independent experiments. The positions of molecular mass markers in kDa are indicated on the left of each immunoblot.

VACV proteins C4 and C16 share ~45% aa sequence identity overall, but this is higher in their C-terminal domains that each bind the Ku proteins (16, 18), whereas their N-terminal domains are more divergent and differ in function. To seek additional functions of C4, an unbiased quantitative proteomics approach was adopted using tandem mass tagging (TMT) and mass spectrometry (MS). For this, HEK293T cells were transfected with plasmids expressing tandem affinity purification (TAP)-tagged C4 or an empty vector (EV), and 16 h later cells were infected with a VACV strain lacking both C16 and C4, v∆C16∆C4 (15, 16). The TAP tag contains two copies of the Strep tag and one copy of the FLAG epitope (34). Cells were then lysed and subjected to streptavidin affinity purification (AP). The eluates were labeled with TMT reagents and were then combined and analyzed by MS. A comparison of C4 with EV is shown in a volcano plot (Fig. 1A). C4 and its known interacting partners, Ku70 and Ku80, were seen as expected, but, interestingly, all three filamins (FLNA, B, and C) were also detected, with FLNB being most abundant (Fig. 1A). ANKHD1, a protein with scaffolding roles, was enriched but was only just above the cut-off for statistical significance, and PRDX1 was of low statistical significance and magnitude. Therefore, ANKHD1 and PRDX1 were not investigated further. Given that the expression of FLNC is mainly linked to muscle cells (25, 26), whereas VACV and some other orthopoxviruses have more epidermal tropism, FLNA and FLNB were prioritized for validation and follow-up.

To confirm the interaction of C4 and FLNs, TAP-tagged C4 and C16 were overexpressed in HEK293T cells, purified with streptavidin beads, and analyzed by immunoblotting. Endogenous FLNB and FLNA co-precipitated with C4, but not with C16, and the enrichment appeared greater with FLNB than FLNA (Fig. 1B). The interaction between C4 and FLNB/A was also confirmed during infection, with the FLNs and C4 expressed at endogenous levels. Cells were infected with vC4-TAP or vC6-TAP in which VACV protein C4 or C6 are fused with a TAP tag and expressed from their natural promoters. C6 is another VACV protein that is expressed early after infection in the cytoplasm. C6 inhibits the activation of IRF3 (35) and the JAK-STAT pathway downstream of type I IFN signaling (36, 37) and also induces the proteolytic degradation of HDAC4, HDAC5, and TRIM5α (36, 38, 39). After the tagged proteins were affinity purified via the strep tag, immunoblotting showed that both FLNA and FLNB co-purified with C4, but not C6 (Fig. 1C).

A reciprocal immunoprecipitation (IP) was then done using HA-tagged FLNs. β-spectrin was used as a cytoskeletal control protein of similar size that also interacts with actin (40, 41). Cells were transfected with plasmids to express these proteins and then were infected with either vC4-TAP (Fig. 1D) or vC6-TAP (Fig. 1E), and an HA IP was performed. C4, but not C6, co-immunoprecipitated with FLNB and, to a much lower degree, with FLNA, whereas β-spectrin did not interact with either viral protein (Fig. 1D and E).

Finally, to determine if C4 interacts with FLNs via its N- or C-terminal domain, cells were transfected with plasmids encoding TAP-tagged C4 mutants that expressed C4 aa 1–156 (C4-N) or aa 167–316 (C4-C) (16). Only full-length C4 and C4-N co-precipitated endogenous FLNB and FLNA (Fig. 1F), showing that C4 aa residues 1–156 are sufficient to co-precipitate the FLNs. So C4 has a distinct function to C16, and this is mediated by its more divergent N-terminal domain.

### C4 associates with FLNB, and FLNA is dispensable for this interaction

In some cases, the interaction of a specific VACV protein with a cellular target can lead to the proteasomal degradation of the host protein (36, 38, 39), whereas in other cases, the cellular protein remains stable, but its function is compromised. Previous quantitative proteomic analysis of human fibroblasts infected with VACV indicated that the levels of FLNB and FLNA were stable during VACV infection (Fig. S1) (38) and so we hypothesized that the C4 interaction observed was somehow beneficial to VACV by altering the function of FLNB or FLNA.

**Fig 2:**
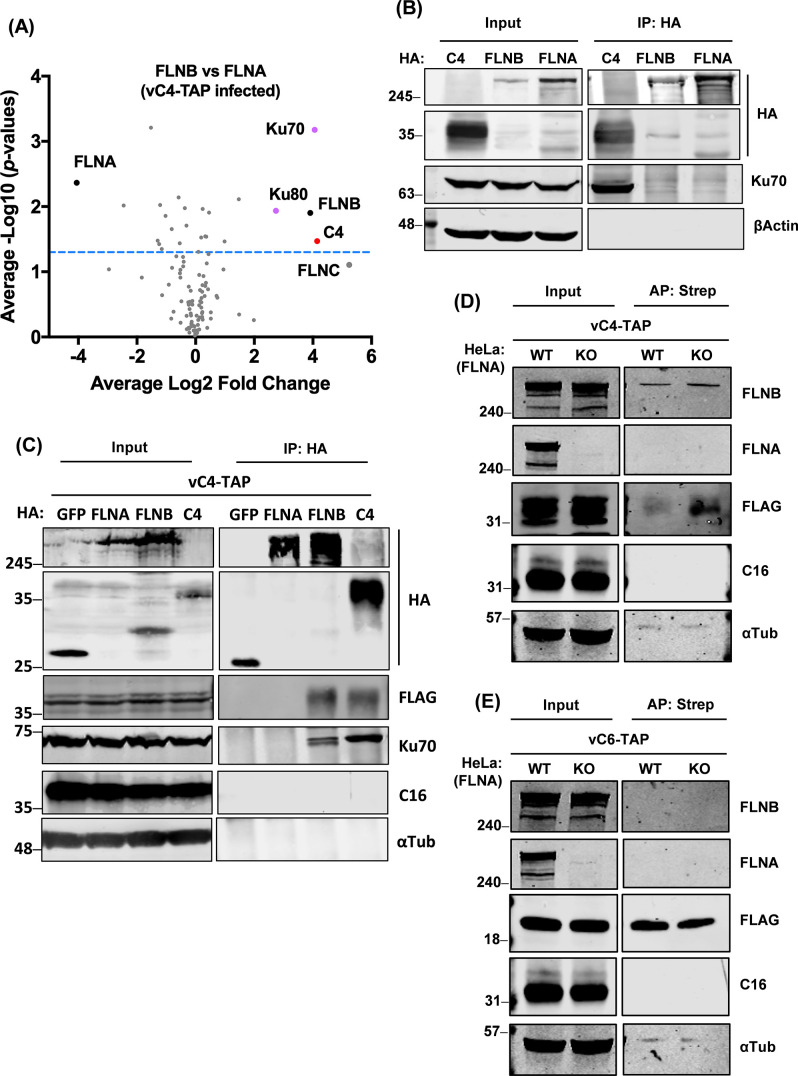
C4 associates with FLNB independent of FLNA. (**A**) HEK293T cells were transfected in triplicate with HA-tagged FLNA, FLNB, or GFP for 16 h and then cells were infected with vC4-TAP at an MOI of 10 for 7 h. Cells were lysed and subjected to HA IP. Clean* Log2 ratios of the mean of three HA-FLNB pulldowns against HA-FLNA pulldown (*x*-axis) are plotted versus −Log10 of the *P*-values (*y*-axis) derived from a Student’s *t*-test. The threshold of *P*-value = 0.05 is indicated with the blue dashed line. FLNA and FLNB baits are shown with black dots, C4 is indicated in red, and C4’s partners Ku70/80 are in purple. FLNC is in gray and was below the level of significance. *Clean: FLNA and FLNB pulldowns were first compared against the GFP pulldown to exclude non-specific hits. (**B**) HEK293T cells were transfected with HA-tagged C4, FLNB, or FLNA, and 22 h later cells were lysed and subjected to HA IP. Proteins were analyzed by immunoblotting. (**C**) HEK293T cells were transfected with HA-tagged GFP, FLNA, FLNB, or C4 and 7 h later cells were infected with vC4-TAP at an MOI of 5 for 16 h. Cells were then lysed, subjected to HA IP, and samples were analyzed by immunoblotting. (**D and E**) Parental (wild-type, WT) HeLa cells were transfected with the PX459 vector containing guide RNA against FLNA to generate FLNA^−/−^ (KO) cells. WT or FLNA^−/−^ HeLa cells were infected with either vC4-TAP (**D**) or vC6-TAP (**E**) at an MOI of 3 for 24 h. Cells were lysed and subjected to AP with anti-streptavidin beads. Proteins were separated by NuPAGE and analyzed by immunoblotting with the indicated antibodies. Data shown are representative of two independent experiments. The positions of molecular mass markers in kDa are indicated on the left of each immunoblot.

C4 co-precipitated FLNB with greater enrichment than FLNA suggesting preferential binding to FLNB, and possibly that FLNA co-purified with C4 as a FLNA-FLNB heterodimer. To address this, a proteomic screen using FLNA or FLNB as bait was done from cells infected with vC4-TAP. HA-tagged FLNA, FLNB, or GFP were expressed by transfection in HEK293T cells, and 16 h later cells were infected with vC4-TAP. Cells were then lysed, and HA-tagged proteins were immunoprecipitated, TMT labeled, combined, and analyzed by MS. The presence of FLNA and FLNB in the corresponding pulldowns versus GFP (Fig. S2) showed efficient IP and MS identification. Interestingly, C4 was undetectable in the FLNA pulldown (Fig. 2A; Fig. S2A) and was only identified as highly enriched in the FLNB pulldown (Fig. 2A; Fig. S2B). Ku70 and Ku80 were also present in the FLNB data set (Fig. 2A; Fig. S2B), but not with FLNA (Fig. 2A; Fig. S2A). Given the abundance of the Ku heterodimer, it was possible that FLNB interacts with the Ku heterodimer independently of C4. To test this, HA-tagged C4, FLNB, or FLNA were expressed by transfection and an HA IP was performed. Only C4 co-precipitated Ku70 (Fig. 2B). This was also tested during infection by expressing HA-tagged GFP, FLNA, FLNB, or C4 followed by infection with vC4-TAP and subsequent HA IP. Endogenous Ku70 was now identified in the FLNB IP, which is attributed to the presence of C4 (Fig. 2C), but it was not pulled down by FLNA or GFP. Notably, HA-tagged C4 co-purified with TAP-tagged C4, indicating C4 dimerization.

To investigate further if FLNA is dispensable for the C4-FLNB interaction, we generated HeLa cells lacking FLNA (KO, FLNA^−/−^) via CRISPR-Cas9 genome editing and validated these by immunoblotting (Fig. 2D) and DNA sequencing, which showed that all FLNA alleles were disrupted and no wild-type (WT) FLNA remained. Wild-type or FLNA^−/−^ HeLa cells were infected with either vC4-TAP (Fig. 2D) or vC6-TAP (Fig. 2E), and then the tagged viral proteins were purified via their strep tag, and their ability to interact with endogenous FLNB and FLNA was investigated by immunoblotting. As expected, FLNB co-precipitated with C4 (Fig. 2D), but not with C6 (Fig. 2E). Notably, C4 could still interact with FLNB in the absence of FLNA (Fig. 2D), which shows that this interaction is independent of FLNA.

### C4 interacts directly with the FLNB C-terminal region

**Fig 3:**
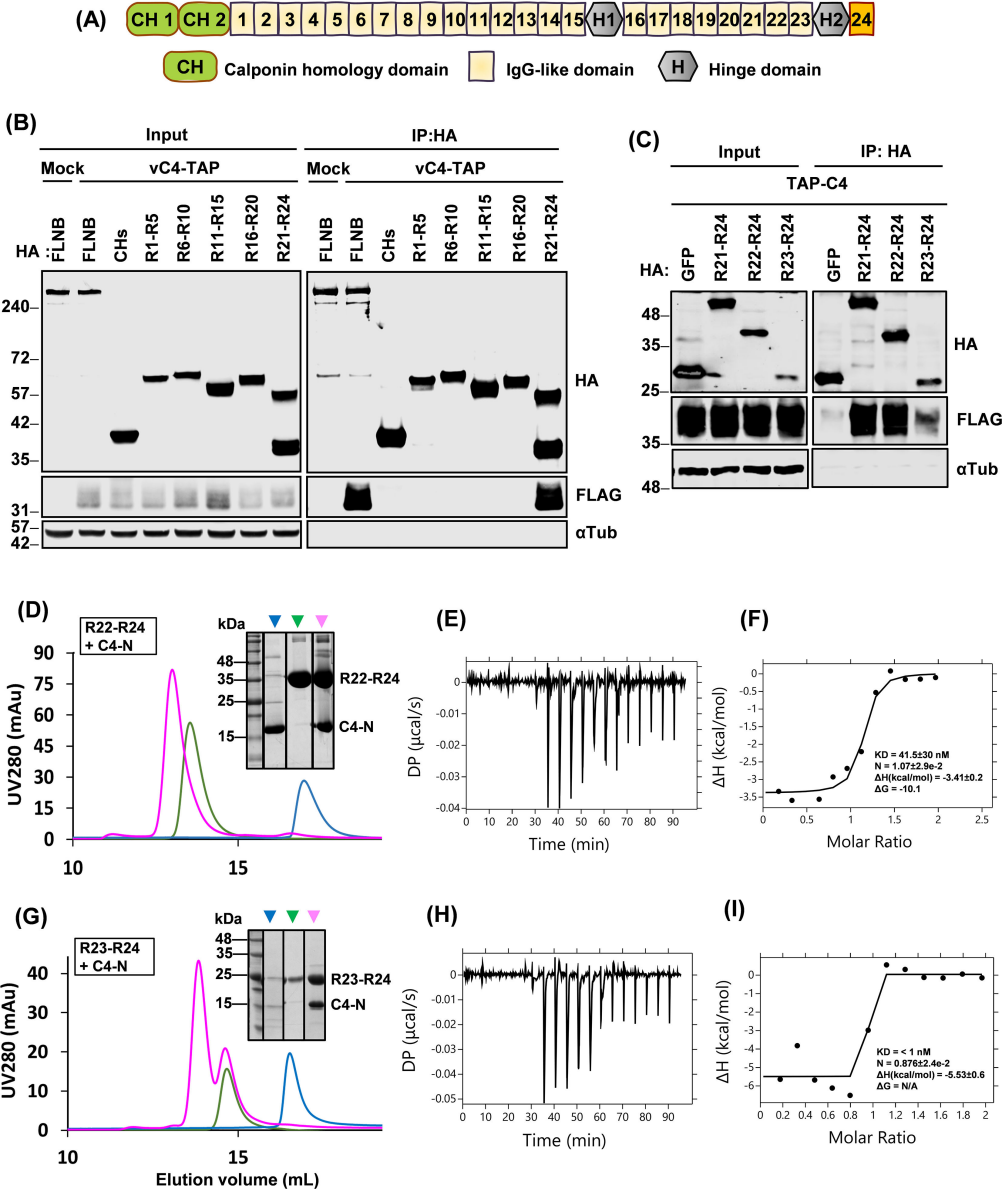
C4 interacts with FLNB via the C-terminal region and this interaction is direct. (**A**) Schematic representation of the FLNB domain structure. (**B**) HEK293T cells were transfected with plasmids encoding HA-tagged full-length FLNB or FLNB truncations for 16 h and then either infected with vC4-TAP at an MOI of 10 or mock-infected for a further 6 h. Cells were lysed and then subjected to HA IP. Proteins were separated by NuPAGE and immunoblotted with the indicated antibodies. (**C**) HEK293T cells were transfected with HA-tagged GFP, or the indicated FLNB truncations, together with TAP-tagged C4 for 24 h. Cells were lysed and subjected to immunoblotting with antibodies against the indicated tags or α-tubulin. (**D**) Analytical size-exclusion chromatography (SEC) for FLNB repeat 22–24 (**R22–R24**) and C4 residues 1–149 (**C4-N**). The elution profiles are shown for R22–R24 (green trace), C4-N (blue trace), or R22–R24 and C4-N mixture (at 1:1 molar ratio, pink trace) from a Superdex 200 Increase 10/300 GL column. Inset image shows the Coomassie-stained SDS-polyacrylamide gel analyses of elution peaks 1–3 in the overlaid chromatograms. The positions of R22-R24 and C4-N are shown. (**E**) Representative isothermal titration calorimetry (ITC) titration curves showing the interaction between C4-N and FLNB R22-24 and baseline-corrected differential power (DP) versus time. (**F**) Binding curve showing integrated changes in enthalpy against molar ratio. (**G**) SEC analysis for FLNB R23-R24 and C4-N. The elution profiles are shown of R23-R24 (green trace), C4-N (blue trace), or R22-R24 and C4-N mixture (at 1:1 molar ratio, pink trace) from a Superdex 200 Increase 10/300 GL column. The inset image shows the Coomassie-stained SDS-polyacrylamide gel. (**H**) Representative of ITC titration curves showing interaction between C4-N and FLNB R23-24, and baseline-corrected differential power (DP) versus time. (**I**) Binding curve showing integrated changes in enthalpy against molar ratio. KD, corresponding dissociation constant; *N*, number of binding sites; Δ*H*, enthalpy change; Δ*G*, change in Gibbs free energy. Data shown are representative of three independent experiments, or (**E**) of an experiment performed in triplicate. The positions of molecular mass markers in kDa are indicated on the left of each immunoblot (**B and C**) or Coomassie-stained SDS-polyacrylamide gel (**D and G**).

Next, we sought to determine which region(s) of FLNB were needed for co-precipitation with C4 and whether the interaction between C4 and FLNB was direct. A collection of HA-tagged FLNB truncation mutants (42) (Fig. 3A and B) were tested for interaction with C4 in vC4-TAP-infected cells. C4 co-immunoprecipitated only with full-length FLNB and the R21–R24 C-terminal fragment of 50 kDa (Fig. 3B). Additional HA-tagged FLNB mutants (R22–R24 and R23–R24) were then constructed to identify the smallest region sufficient for this interaction. These FLNB fragments and R21–R24 were co-expressed with C4-TAP by transfection, and all were found to co-precipitate with C4, whereas GFP did not (Fig. 3C).

To determine if C4 and FLNB interact directly and, if so, which part of FLNB is essential for this, C4-N and FLNB fragments (R22–R24, R23–R24, R22–R23, and R23) were expressed in *Escherichia coli*, purified, and tested for interaction with C4-N by analytical size exclusion chromatography (SEC). The elution profiles of C4-N and FLNB R22–R24 (Fig. 3D) and C4-N and FLNB R23–R24 (Fig. 3G) changed when both proteins were present, indicating that C4-N formed a complex with both R22–R24 and R23–R24 fragments. Isothermal titration calorimetry (ITC) showed that both complexes were formed at equimolar concentrations (Fig. 3E, F, H, and I). The estimated dissociation constants (Kd) for R22–R24 and R23–R24 with C4-N are in nanomolar (41.5 ± 30 nM) and sub-nanomolar (<1 nM) range, respectively, suggesting that C4 has a slightly higher affinity for R23–R24 than for R22–R24. An accurate measurement of the affinity between C4 and R23–R24 was not possible, because unbound C4 rapidly formed a complex upon mixing with R23–R24, and the titration data could not be fitted using a single-site binding mode. Due to limitations of instrument sensitivity, using lower concentrations of C4 and FLNB resulted only in noisier data without improving accuracy. Overall, these data suggest that R23–R24 has the essential binding site for C4, and the presence of R22 in the longer fragment may influence this interaction. Additional SEC analyses for C4-N and R22–R23 or R23 alone showed no interaction (Fig. S3), indicating that the H2-R24 domain is essential for the formation of the C4/FLNB complex.

### FLNB dimerization is not required for its interaction with C4

**Fig 4:**
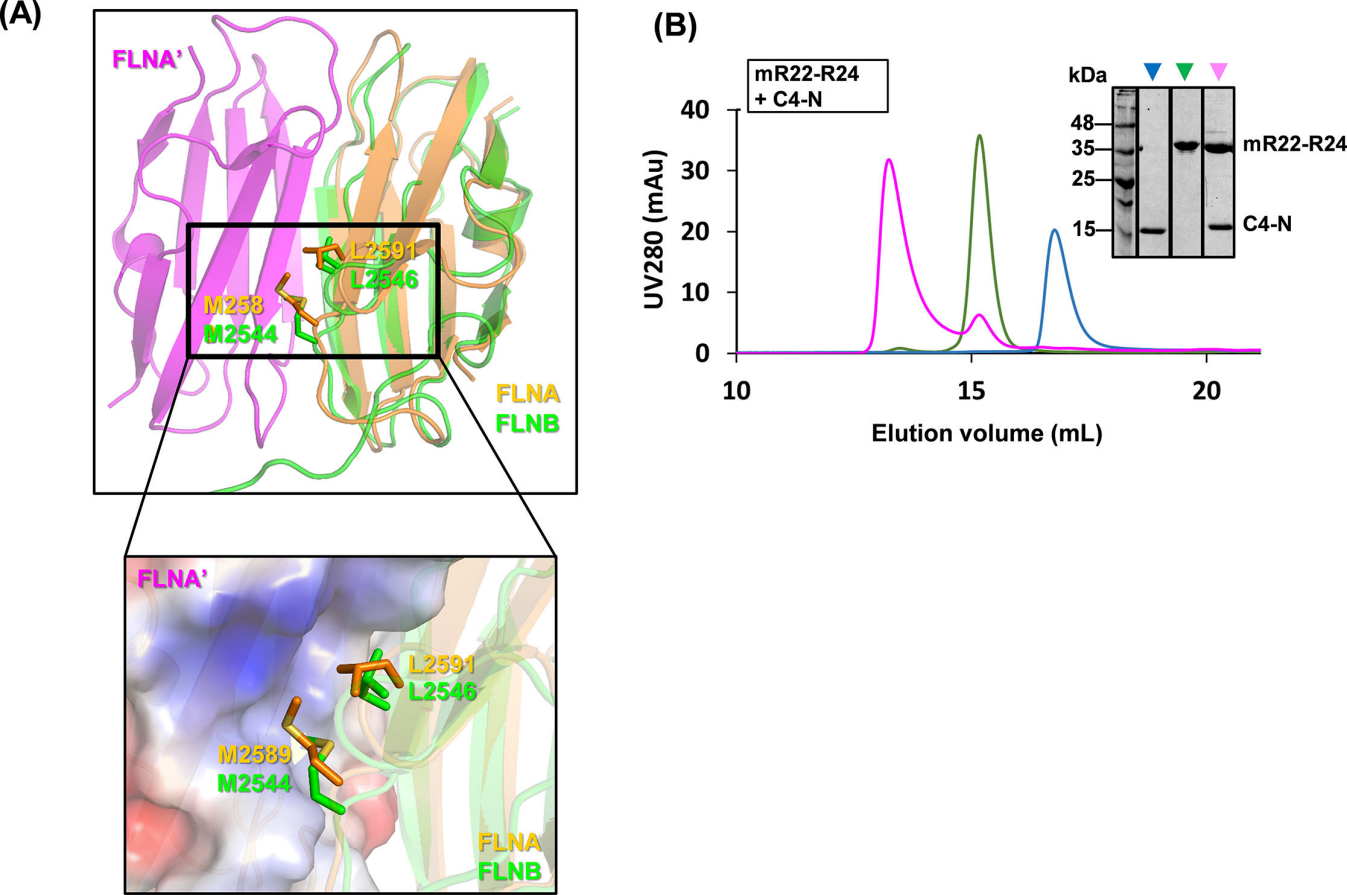
FLNB dimerization is not required for its interaction with C4. (**A**) A structural model of FLNB R24 predicted by RaptorX (green) aligned to chain A (orange) of the FLNA dimer structure (PDB code 3CNK). Two key hydrophobic residues, M2589 and L2591, at the dimer interface of FLNA (orange) and their equivalent residues, M2544 and L2546, in FLNB (green) are shown as sticks. Below is a close-up view of the dimer interface showing the hydrophobic residues from FLNB and FLNA, chain A (FLNA) packing against a complementary pocket on the surface of FLNA, chain B (FLNA). The surface of FLNA is colored by surface potentials: red and blue denote positive and negative charges, respectively, while white areas denote non-charged surfaces. (**B**) Analytical size-exclusion chromatography analysis for an FLNB dimerization-deficient mutant R22–R24 (mR22–R24) and C4-N. The inset image shows the Coomassie-stained SDS-polyacrylamide gel of elution peaks in the overlaid chromatograms. mR22–R24 and C4-N positions are indicated. Data shown are representative of three independent experiments. In panel **B,** the positions of molecular mass markers in kDa are indicated on the left.

The FLNB R24 domain is known to mediate FLNB dimerization (43). However, the structure of the FLNB R24 dimer is lacking. To investigate whether dimerization is essential for the interaction with C4, the structure of FLNB R24 monomer (PDB code 2EED) was first aligned to the structure of chain A of FLNA R24 dimer (PDB code 3CNK) with a root mean square deviation of 1.066 Å across 86 residues (Fig. 4A). Two hydrophobic residues, M2589 and L2591, from FLNA chain A, are found to make significant hydrophobic contact by protruding into a surface pocket on chain B. The two equivalent residues in FLNB are M2544 and L2546, respectively. Therefore, two mutations M2544E and L2546D were introduced into the predicted dimerization interface of FLNB R22–R24 in an attempt to generate a dimerization-deficient mutant (mR22–R24). Analytical SEC studies show mR22–R24 could no longer form dimers, as shown by a rightward shift of its elution peak (Fig. 4B green peak compared to Fig. 3D green peak). Nevertheless, mR22–R24 was still able to bind C4 and form a complex (Fig. 4B, pink peak), suggesting dimerization is not a prerequisite for C4 binding.

### The C4 N-terminal domain contributes to virulence

**Fig 5:**
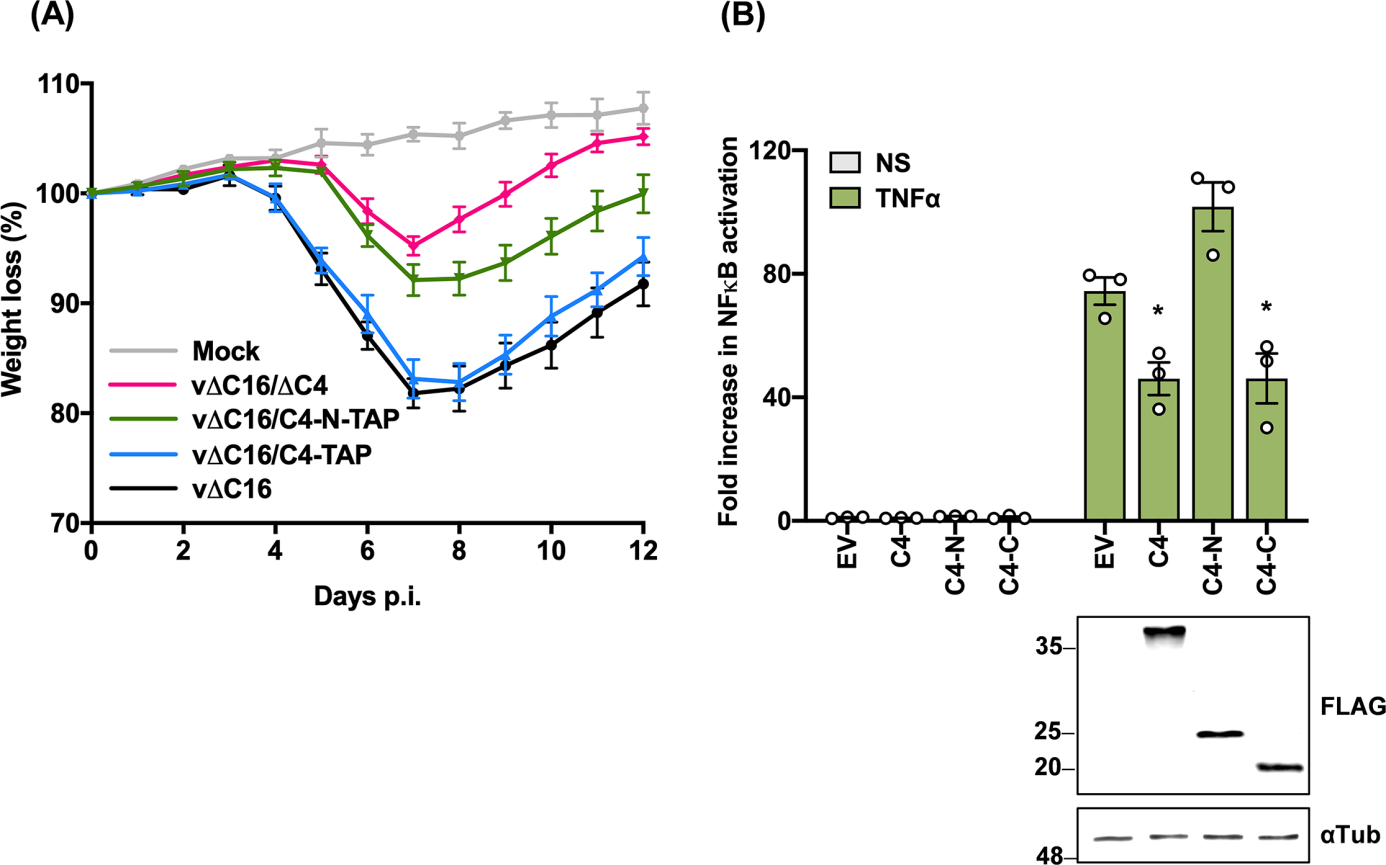
The C4 N terminus contributes to virulence. (**A**) BALB/c mice (*n* = 5) were infected i.n. with 1 × 10^5^ p.f.u./mouse of the indicated viruses and their weights were measured daily. Data are expressed as a percentage of the mean weight of the same group of animals on day 0 ± SEM. Data shown are representative of two independent experiments. (**B**) HEK293T cells were co-transfected in triplicate with a firefly luciferase reporter plasmid under the control of an NF-κB-dependent promoter, a *Renilla* luciferase transfection control plasmid (7 ng/well), and 60 ng/well of TAP-tagged C4, C4 aa 1–156 (**C4-N**), or C4 aa 167–316 (**C4-C**), or EV as a control for 24 h. Cells were then stimulated with TNFα for 8 h or mock-stimulated. Cells were harvested in passive lysis buffer, and firefly luciferase activity was measured and normalized to the *Renilla* luciferase activity. Then, each sample was compared to the average value of EV control to obtain the fold increase. An immunoblot with antibodies against FLAG-tagged C4 proteins and α-tubulin is shown below. The positions of molecular mass markers in kDa are shown on the left of the immunoblots. Data shown are representative of at least three independent experiments. Statistical significance was measured by a Student’s *t*-test. *P ≤ 0.05. Error bars represent the standard error of the mean.

Although C4 interacts with FLNB via its N-terminal domain, the biological significance of this is unknown. To address this, a recombinant VACV was generated, which expressed only the N-terminal domain of C4, and the virulence of this virus was tested in a mouse model. Given the sequence similarity of C4 with C16, this virus was constructed in ∆C16 background to eliminate any redundancy that might mask possible phenotypes. The N-terminal region (aa 1–156) of C4 was inserted into v∆C16/∆C4 virus by transient dominant selection (Materials and Methods). The genomic structure of the resultant virus, called v∆C16/C4-N-TAP, was confirmed by PCR of the C4 locus (Fig. S4). BALB/c mice were infected intranasally (i.n.) with this virus and compared to parallel infections with v∆C16 (17), v∆C16/C4-TAP, and v∆C16/∆C4 (16). The weights of individual mice were measured daily and compared to the weights on day 0. Infection with all the viruses resulted in weight loss, and this was greatest when C4 or C4-TAP were present (black and blue lines) and least when C4 was absent (pink line), showing C4 contributes to virulence as noted previously (15). In comparison, a virus expressing just the C4-N domain caused intermediate weight loss, showing that the C4-N terminal domain contributes to virulence (Fig. 5A). Since the virulence of this virus was intermediate between viruses expressing full-length C4 or lacking C4, these data also indicate that, in the absence of C16, the C-terminal Ku-binding domain of C4 also contributes to virulence.

In addition to binding Ku and thereby blocking DNA sensing (16), and binding to FLNB, C4 also inhibits NF-κB activation (15). To explore which domain of C4 mediates this inhibition, a reporter gene assay measuring NF-κB activation was undertaken with full-length C4, C4-N, or C4-C (Fig. 5B), and immunoblotting was used to confirm protein expression. Following the addition of TNFα, the level of NF-κB activation was reduced to a similar degree by full-length C4 and C4-C, but not C4-N, suggesting that C4-N is not involved in the reduction of NF-κB signaling.

### C4 does not affect the MAPK signaling pathway

**Fig 6:**
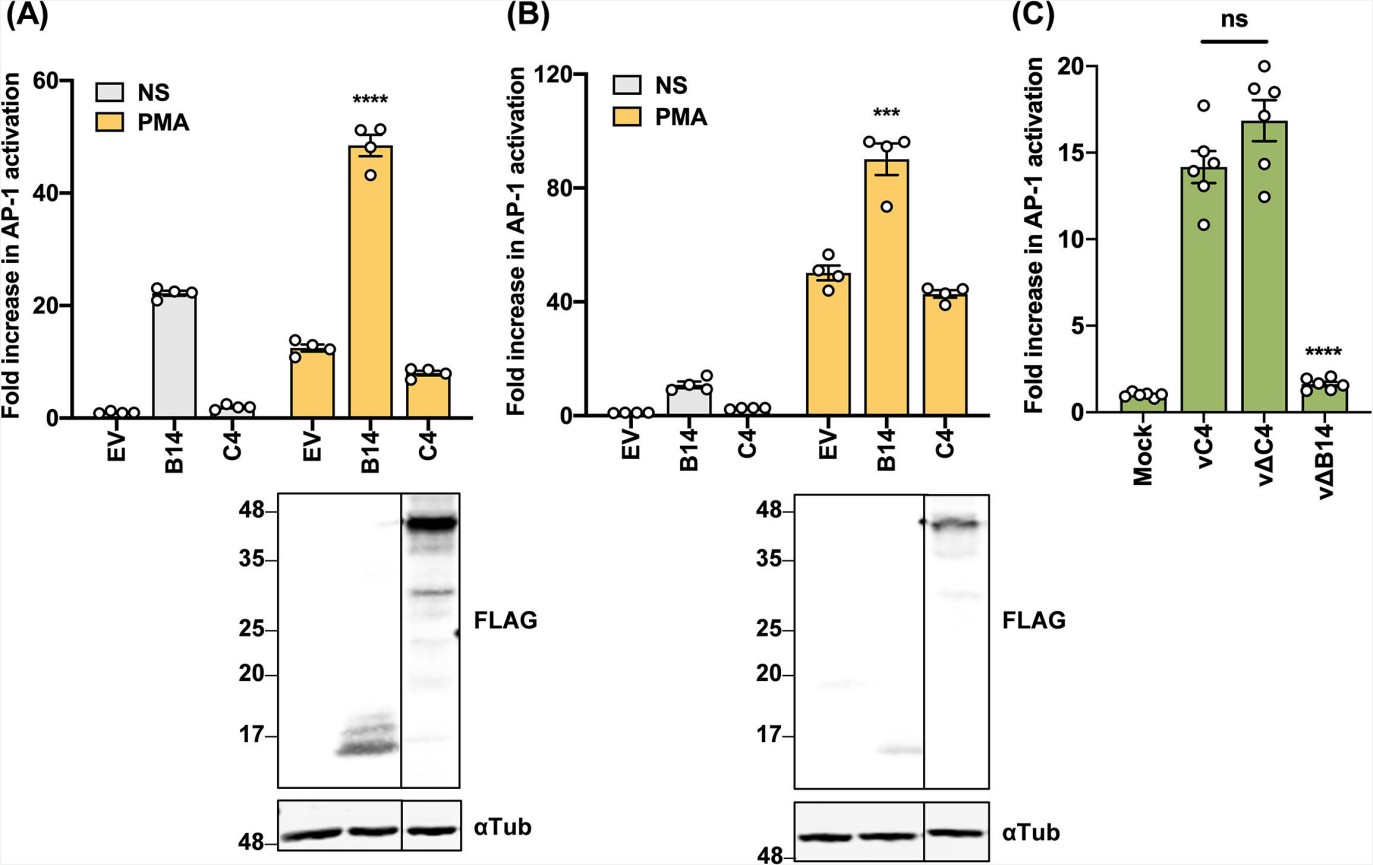
C4 does not affect MAPK signaling. (**A**) HEK293T cells or (**B**) HeLa cells were co-transfected in quadruplicate with a reporter plasmid encoding firefly luciferase under the control of the AP-1 promoter (AP-1-Luc), a *Renilla* luciferase transfection control plasmid (10 ng/well), and 100 ng/well of B14-FLAG or C4-TAP for 24 h. Cells were then stimulated with phorbol 12-myristate 13-acetate (PMA) or left unstimulated for 24 h. Cells were harvested in passive lysis buffer, and firefly luciferase activity was measured and normalized to the *Renilla* luciferase activity. Each sample was compared to the average value of EV control to obtain the fold increase. An immunoblot against FLAG and α-tubulin is shown below, and the positions of molecular mass markers are shown in kDa on the left. (**C**) HeLa cells were co-transfected in sextuplicate with AP-1-Luc and TK-*Renilla* reporters as in panel **A**, and 24 h later cells were mock-infected or infected with the indicated viruses at an MOI of 10 for 16 h. Data shown are representative of two independent experiments. Statistical significance was measured by a Student’s *t*-test. ns, non-significant, ***P ≤ 0.001, and ****P ≤ 0.0001. Error bars represent the standard error of the mean.

FLNB interacts with MAP3K1, MAP2K4, and c-Jun N-terminal kinase (JNK) and so acts as a scaffold to bring these components together and facilitate JNK activation, independent of its actin-binding ability (33). Additionally, ISG15 acts as a negative regulator of this function by binding to FLNB and blocking its binding to MAP3K1 and MAP2K4 (44). To test if C4 interacts with FLNB to modify MAPK signaling, an AP-1-luciferase plasmid (AP-1-Luc) was used in reporter gene assays in both HEK293T (Fig. 6A) and HeLa (Fig. 6B) cells. VACV protein B14, a small, Bcl-2-like protein, activated the pathway as reported (45), whereas C4 did not affect AP-1 activation in either cell line (Fig. 6A and B). AP-1 activation was also tested following infection with WT VACV (vC4) or a VACV lacking C4 (v∆C4) or B14 (v∆B14). As reported, VACV infection activated AP-1 signaling and this was largely due to the B14 protein (45), but this was unaffected by the loss of C4 (Fig. 6C). Overall, in the conditions tested, C4 does not affect the MAPK signaling pathway, indicating that its interaction with FLNB may affect a different function of FLNB.

### FLNB affects VACV growth and spread in human and mouse cell lines

**Fig 7:**
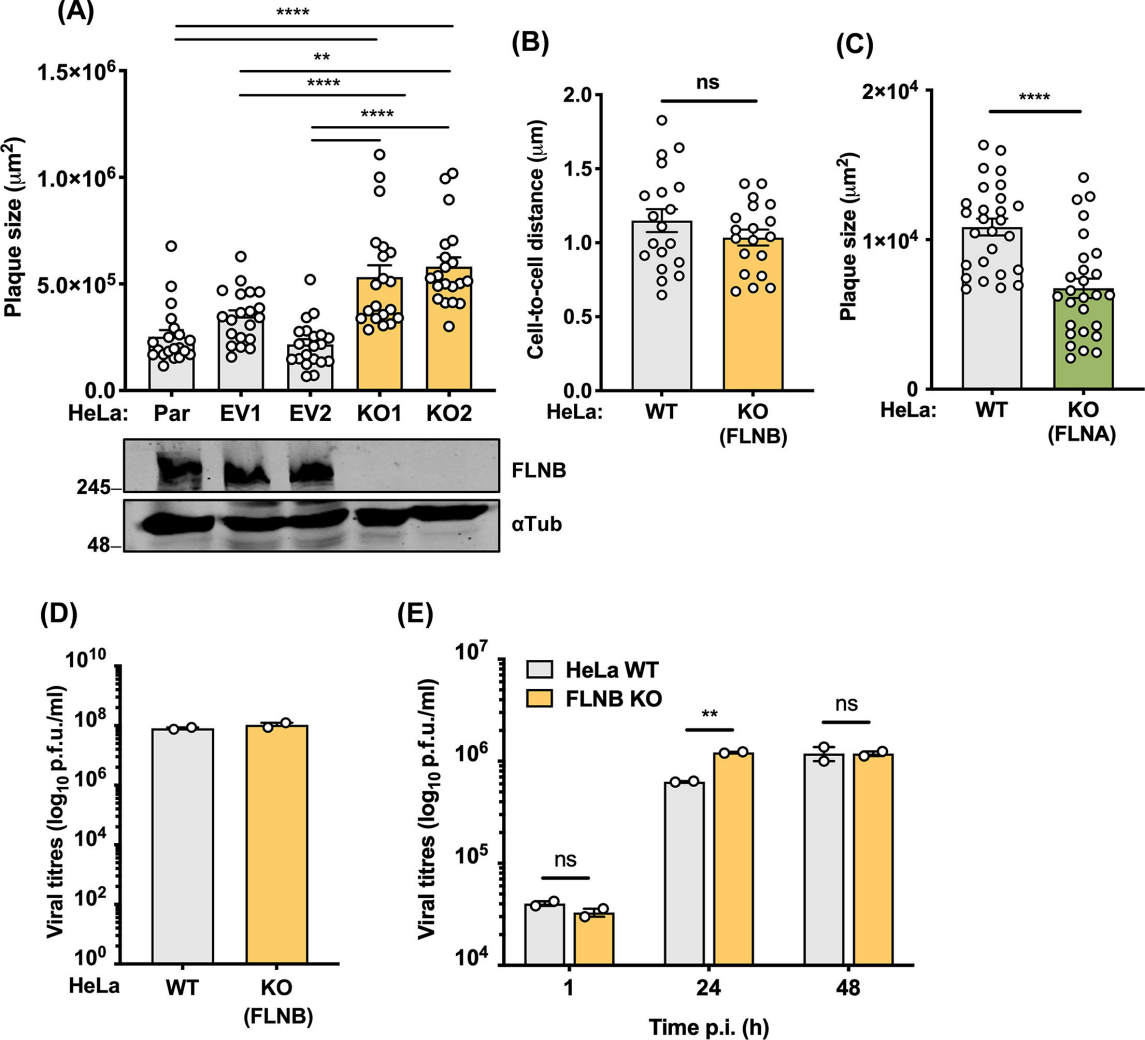
Enhanced VACV plaque size and virus titers in human FLNB^−/−^ cell lines. (**A**) Parental HeLa cells were transfected with either an empty PX459 vector (EV) or the PX459 containing guide RNA against FLNB to generate the FLNB^−/−^ cells. Single-cell clones of EV and FLNB^−/−^ cells were then selected. Cell monolayers of HeLa Par cells, two EV, and two FLNB^−/−^ clones were infected with VACV A5-GFP to give well-separated plaques. After 2 days, images of GFP-expressing plaques were recorded and quantified by ImageJ software (*n* = 20 per condition). Lower panel: immunoblot analyzing the levels of FLNB and α-tubulin. The positions of molecular mass markers in kDa are shown on the left. (**B**) Distances between adjacent nuclei of FLNB WT and FLNB^−/−^ HeLa cells grown in cell monolayers (*n* = 19). (**C**) Monolayers of HeLa WT or FLNA^−/−^ cells were infected with VACV A5-GFP to give distinct plaques. After 2 days, images of plaques were recorded as in panel **A,** and plaque area was determined by ImageJ software (*n* = 27 per condition). (**D**) WT and FLNB^−/−^ HeLa cells were infected with vC4-TAP at an MOI of 5. Infected cells and supernatants were collected at 24 hpi, combined, and the infectious virus titers were determined by plaque assay on BS-C-1 cells. (**E**) WT and FLNB^−/−^ HeLa cells were infected with vC4-TAP at an MOI of 0.01 for the indicated times. Virus titers were determined as in panel **D**. Data shown are representative of at least two independent experiments. Statistical significance was measured by a Student’s *t*-test. ns, non-significant, **P ≤ 0.01, ***P ≤ 0.001, and ****P ≤ 0.0001. Error bars represent the standard error of the mean.

To investigate the potential role of FLNB during VACV infection, we knocked out the *flnb* gene from HeLa cells using CRISPR-Cas9 genome editing and validated two knockout cell lines via immunoblotting (Fig. 7A) and DNA sequencing. The parental (Par) cell line and two control lines made in parallel using an EV (see Materials and Methods for details) displayed the expression of the native FLNB, whereas the FLNB^−/−^ cell lines did not. DNA sequencing showed that in FLNB KO1 and KO2 cell lines, only mutant alleles were detected in which the reading frame was disrupted by frameshift or nonsense mutation and that no WT allele remained. The monolayers of these cells were then infected with a VACV strain that expresses GFP fused to the capsid protein A5 (46), and the plaque size was measured 2 days later. FLNB^−/−^ cells developed larger plaques than the WT cells (Fig. S5), and plaque size measurements revealed a significant increase in plaque size in both FLNB^−/−^ cell lines compared to the polyclonal parental line and the two control clones (EV1-2) (Fig. 7A). FLNB is a cytoskeletal protein with an important role in cellular architecture and deleting it from cells could affect cell size or morphology, and thereby the size of virus plaques formed. However, both HeLa FLNB^−/−^ cell lines had the same size as control cells, as judged by measuring the distance from one nucleus to the next on cell monolayers (Fig. 7B; Fig. S6). This is also consistent with prior studies in human fibrosarcoma cells indicating that FLNB depletion does not affect cell spreading (47).

**Fig 8:**
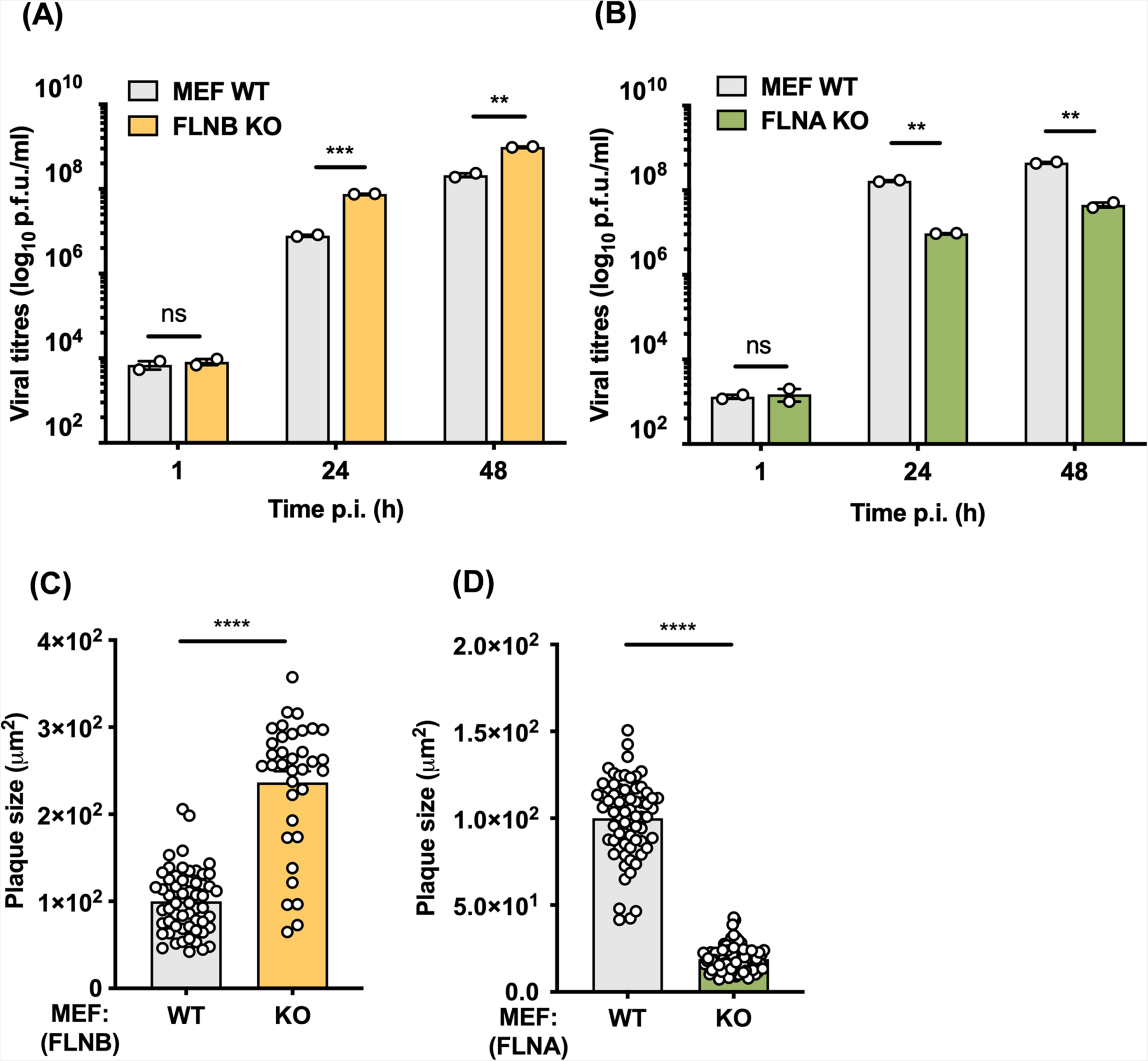
VACV infection of FLNB^−/−^ MEFs, but not FLNA^−/^− MEFs, resulted in increased virus titers and plaque size. (**A**) WT or FLNB^−/−^ MEFs and (**B**) WT or FLNA^−/−^ MEFs were infected with vC4-TAP at an MOI of 0.01, and cell-associated virus was collected at the indicated times and titrated by plaque assay on BSC-1 cells. (**C**) Cells from panel **A** and (**D**) cells from panel **B** were infected with VACV A5-GFP for 2 days until well-separated plaques had formed. Images of GFP-expressing plaques were recorded and quantified by ImageJ software (minimum *n* = 35 plaques per condition). Data shown are representative of at least two independent experiments. Statistical significance was measured by a Student’s *t*-test. ns, non-significant, **P ≤ 0.01, ***P ≤ 0.001, and ****P ≤ 0.0001. Error bars represent the standard error of the mean.

To determine if the increased virus plaque size was specific to FLNB, VACV plaque size was also measured in HeLa FLNA^−/−^ cells generated in the same way. In these cells, VACV formed significantly smaller plaques than in WT cells (Fig. 7C; Fig. S7), indicating that the phenotype observed in the FLNB^−/−^ cells is specific to FLNB. Next, we measured the yield of infectious virus in FLNB^−/−^ cells compared to WT controls after infection at high MOI (MOI = 5) with vC4-TAP for 24 h or low MOI (MOI = 0.01) at 1, 24, and 48 h post-infection (hpi). The yield of the total virus (cell-associated and extracellular virus combined) was determined by plaque assay. No difference in virus titers was observed after high MOI (Fig. 7D); however, a statistically significant increase in virus titers was detected at 24 h after low MOI in the FLNB^−/−^ cells compared to the WT (Fig. 7E). To investigate if these differences in human cells were also seen in mouse cells, mouse embryo fibroblasts (MEFs) lacking either FLNA or FLNB were obtained, and the yield of virus and size of plaques formed on these cells were measured. A significant increase in VACV titer was observed in FLNB^−/−^ MEFs derived from FLNB null mice (48) after low MOI with vC4-TAP at 24 and 48 hpi (Fig. 8A). In contrast to FLNB, VACV infection in FLNA^−/−^ MEFs (derived from FLNA^−/−^ mice) resulted in significantly reduced viral titers (Fig. 8B). As seen in HeLa cells, in FLNB^−/−^ MEFs, virus plaque size was significantly increased compared to control, whereas in FLNA^−/−^ MEFs, the plaque size was reduced (Fig. 8C and D, respectively). This indicates a proviral role for FLNA, whereas FLNB is antiviral, and this might explain why VACV protein C4 has evolved to target FLNB rather than FLNA. In summary, deletion of FLNB, but not FLNA, in both human and mouse cells promotes VACV spread, indicating that FLNB has antiviral activity against VACV.

## DISCUSSION

Although VACV encodes a high number of immunomodulatory proteins (1), it still makes efficient use of its coding capacity, and many proteins have multiple functions. Examples include protein C6 that diminishes the activation of IRF3 and IRF7 (35), inhibits type I IFN-induced signaling (37), and induces degradation of HDAC4 and HDAC5 (36, 38); protein E3 that has distinct N and C terminal domains to bind Z-nucleic acid (49, 50) or dsRNA (51), respectively (52); and proteins C16 and C4, the subject of this study. C4 and C16 are each intracellular proteins that are expressed early during infection and are non-essential for virus replication in cultured cells (15, 17). Although related, C4 and C16 have non-redundant functions because loss of either protein causes virus attenuation despite the retention of the other (15, 17), and loss of both proteins causes further attenuation (16). The conserved C-terminal domains of these proteins each bind the Ku proteins to inhibit dsDNA sensing (16, 18). C4 also inhibits NF-κB activation (15), and this function is shown here to also reside in the C-terminal domain. Although the N-terminal domain of C16 binds PHD2 and induces a hypoxic response (22), this function is not shared by C4 (16) and no role for the C4 N-terminal domain was known.

To address the function of the C4 N-terminal domain, we undertook an unbiased proteomic search for C4-binding partners from VACV-infected cells and identified the cytoskeletal proteins FLNA/B/C, the scaffolding protein ANKHD1, and the peroxidase PRDX1 (Fig. 1A). Only FLNB and FLNA were investigated further, given the low *P*-values for the co-purification with ANKHD1, PRDX1, and FLNC. The interaction of C4 with FLNB was validated by reciprocal immunoprecipitations (Fig. 1B through E), mapped to the C4 N-terminal domain (Fig. 1F), shown to be independent of FLNA (Fig. 2D and E), and to be direct (Fig. 3D). Mapping of the region of FLNB needed for the interaction identified the C-terminal region spanning Ig-like repeats 23–24 and including the hinge 2 region (Fig. 3B and C). Biophysical measurements indicated that C4 binding to FLNB is of very high affinity (Fig. 3E, F, H, and I), suggesting that C4 might outcompete other proteins that bind FLNB via this region. The identification and mutagenesis of FLNB residues needed for FLNB dimerization did not abrogate binding to C4 (Fig. 4), so binding is independent of dimerization. An interesting observation was that C4 itself can dimerize (Fig. 2C), and similarly, a recent structural study demonstrated that the C-terminal domain of C16 can dimerize (20). However, C4 and C16 do not interact (16) and thus can perform their functions independently.

MS data indicated that C4 associates preferably with FLNB, and only low amounts of FLNA were identified. Since FLNs can form heterodimers, FLNA could be pulled down indirectly via FLNB, although in the FLNA pull-down MS data set, there was no trace of FLNB, and recombinant FLNA is unable to heterodimerize with either FLNB or FLNC (43).

FLNs share about 70% aa identity, with their hinge regions being most divergent (30, 31). So, we wondered if C4 interacts with FLNB hinge 2; however, this could not be tested because attempts to express H2 or R24 alone were unsuccessful. Additional studies, such as SEC with multi-angle light scattering (SEC-MALS) or analytical ultracentrifugation (AUC), are needed to determine the binding stoichiometry between C4 and FLNB, and a high-resolution structure of their complex will be useful to elucidate the mechanism of interaction. FLN dimerization is crucial for crosslinking actin (53) but not for C4 binding, so it is possible that C4 binds FLNB to reduce FLNB dimerization or has other functions.

The new demonstration that the N-terminal domain of C4 binds FLNB adds to the functions of C4. This region of C4 also contributes to virus virulence because a mutant VACV expressing only the C4 N-terminal domain had a virulence intermediate between that of viruses lacking C4 or expressing WT C4 (Fig. 5A). C4 is a multi-functional protein and also inhibits NF-κB activation (15) and DNA sensing (16); however, these other functions are mediated by the C-terminal domain (Fig. 5C) (16). It is possible that the C4 N-terminal domain contributes to virulence via binding FLNB, but this will require confirmation using C4 mutants that have lost binding to FLNB while retaining other known functions.

FLNB is an actin-binding protein that assists in the formation of three-dimensional filamentous actin networks and acts as a scaffold for receptor activation and signal transduction (32). It is also involved in several other cellular functions including adhesion, proliferation, motility, and membrane stability, and these are performed through a large variety of different mechanisms (47, 54). Interestingly, mutations across the FLNB sequence have been linked to severe skeletal disorders (55). In other studies, FLNB was found to have non-cytoskeletal roles, such as being an RNA-binding protein (56) and functioning in alternative splicing and transcription (57). FLNB also has a role in innate immune signaling by acting as a scaffold for mitogen-activated protein kinases in response to type I IFN sensing (33). However, C4 was found not to affect this pathway (Fig. 6). FLNB also interacts with the hepatitis B core protein and this enhances viral replication (58). Here, we show that VACV infection on FLNB^−/−^ cells resulted in an increase in plaque size (Fig. 7; Fig. S5) and a statistically significant increase in viral titers in both human and murine cells (Fig. 7E and 8A). This indicates that FLNB is anti-viral and restricts poxviruses. In contrast, FLNA^−/−^ cells showed the opposite phenotype, with reduced VACV plaque size (Fig. 7C and 8D; Fig. S7) and lower viral titers (Fig. 8B). This is consistent with prior reports that FLNA is pro-viral (59–61), although additional studies with different viruses suggest an anti-viral role (62, 63). The interaction of C4 with FLNB seems unrelated to the exploitation of actin- and tubulin-based motility during VACV infection, because these processes occur normally in the absence of C4. A study suggested that cortical actin may provide a barrier to VACV egress (64) and so, given the interaction of FLNA and FLNB with actin, the loss of FLNA or FLNB might influence the ease of transport of virus particles across cortical actin either during the entry of incoming virions or the egress of nascent virions. However, if this were so, it is unclear why the influence of FLNA and FLNB would be so different. The exact role of FLNB during VACV infection and the mechanistic consequence of C4 binding to it will require additional investigation that is beyond the scope of this study.

Altogether, we have discovered that the C4 N-terminal domain contributes to virulence and binds directly to the C-terminal region of FLNB. This study also shows that FLNB has an anti-poxviral role, which may explain why VACV has evolved a protein to target it. Additional studies are needed to investigate how FLNB is anti-poxviral and how the direct interaction of C4 with FLNB may modulate FLNB function. The interplay between pathogens and the cytoskeleton continues to reveal complex and diverse biology and this study contributes to this field.

## MATERIALS AND METHODS

### Cell lines

HeLa (ATCC CCL-2), HEK293T (ATCC CRL-11268), BS-C-1 (ATCC CCL-26), CV-1 (ATCC CCL-70), and MEFs were grown in Dulbecco’s modified Eagle’s medium (DMEM, Life Technologies) supplemented with 10% heat-inactivated fetal bovine serum (FBS, Pan Biotech), or 15% FBS for MEFs, and penicillin-streptomycin (50 µg/mL; Gibco). FLNA^−/−^ or FLNB^−/−^ MEFs together with their controls (FLNA and FLNB WT, respectively) were a gift from Levent Akyurek (University of Gothenburg, Sweden) (48). For the generation of HeLa FLNA^−/−^ and FLNB^−/−^ cells, clonal parental cells were transfected with either the empty PX459 vector (Addgene; plasmid #62988) or the same vector containing guide RNA against FLNA or FLNB exon 3, respectively. The process that was followed was described (65). The genotype of selected FLNA^−/−^ and FLNB^−/−^ clones was verified by DNA sequencing, and this confirmed that all alleles had either frameshift or nonsense mutations and that no WT allele remained (see section “CRISPR/Cas9-mediated genome editing” below for more details and the guide RNA sequences).

### Mice

Female BALB/c mice 6–8 weeks old (*n* = 5) were infected intranasally with the indicated viruses as described (66) following the regulations of The Animals (Scientific Procedures) Act 1986. The weight of each animal was measured daily and compared to the weight of the same animal on day 0.

### Expression vectors

Plasmids expressing codon-optimized C4-TAP and C16-TAP (15, 18) or truncations of C4 (C4-N and C4-C) (16) or a pcDNA4.1-TO expression plasmid expressing FLAG-tagged B14 (45) were described. The TAP tag used in this study consists of Strep and FLAG tags as described (34). pCMX-1H plasmids expressing full-length human FLNB (Uniprot O75369) and the truncations CHs, R1–R5, R6–R10, R11–R15, R16–R20, R21–R24, all with an N-terminal HA tag, were a gift from Hung-Ying Kao (Case Western Reserve University, USA). The FLNB truncations R22–R24 (residues 2,282–2,601) and R23–R24 (residues 2,379–2,601) were amplified by PCR from full-length FLNB using primers: Fwd (R22–R24) 5′-GAG CGG CCG CGG CAC CCT CCG ACG ACG CCC-3′; Fwd (R23–R24) 5′-GAG CGG CCG CGG GAC AAG CGG GGA ACC CTG CCC-3′; and Rv 5′-CCC TCT AGA TTA AGG CAC TGT GAC ATG AAA AGG GCT G-3′. The R21–R24 sequence (residues 2,188–2,601) was re-cloned with Fwd 5′-GAG CGG CCG CGC TTG GTG AAG GAG GCG CCC ACA AGG-3′ and the same Rv primer as above to generate a plasmid encoding a 50-kDa protein. All primers contain the restriction sites *Not*I and *Xba*I that were used for cloning the PCR products. The plasmid encoding full-length human FLNA with an N-terminal HA tag was constructed by amplifying the FLNA open reading frame from an untagged plasmid that was a gift from Stephen Robertson (University of Otago, New Zealand) and cloning the PCR product into pcDNA4/TO-HA with *Not*I and *Xba*I restriction enzymes. The primers used for PCR amplification were 5′-ATA TCG CGG CCG CGA GTA GCT CCC ACT CTC GG-3′ and 5′-ATA CTT CTA GAT CAG GGC ACC ACA ACG CGG TA-3′ containing the restriction sites. A plasmid expressing an N-terminal HA-tagged GFP was constructed by the insertion of the GFP gene (Uniprot P42212) into pcDNA3 (Addgene). A plasmid expressing β2-spectrin was purchased from Addgene (plasmid #31070).

For protein expression in *E. coli*, codon optimized C4 (VACWR024 gene, Uniprot P17370) N-terminal residues 1–149, human FLNB C-terminal fragments R22–24 (residues 2,282–2,601), R22–23 (residues 2,282–2,471), R23–24 (residues 2,379–2,601), R22 (residues 2,282–2,375), and R23 (residues 2,369–2,471) were subcloned into pOPTH vector with an N-terminal His6 tag. To generate the FLNB R22-24 dimerization-deficient mutant (mR22–24), the quick-change mutagenesis PCR (Agilent) was used according to the manufacturer’s instructions.

### Vaccinia viruses

All VACVs used were based on VACV strain Western Reserve. Recombinant VACV (rVACV) strains vC6-TAP (67), vC4-TAP (15), vΔC16 (17), vΔC16ΔC4 (16), and VACV A5-GFP expressing GFP fused to the capsid protein A5 (46) were described. Additional rVACVs were constructed using transient dominant selection (68). For the construction of vΔC16/C4-TAP, oligonucleotides 5′-ATC GAA GCT TGC ACA CAT AAT CCA TTC TCT C-3′ (left arm, LA) and 5′-CGC GAA TTC TCC GAT CCA CCC ACG TGT CAG AT-3′ (right arm, RA), containing restriction sites *Hin*dIII and *Eco*RI, were used in a PCR using pUC13-C4TAPRev plasmid (15) as a template. This amplified the *C4L* ORF with a C-terminal TAP tag and 300 bp of the left flanking region and 303 bp of the right flanking region (Fig. S4). This DNA fragment was cloned into plasmid pUC13-EcoGPTEGFP containing *E. coli* guanylphosphoribosyl transferase (*EcoGPT*) fused in frame with the enhanced green fluorescent protein (*EGFP*) (69). The resultant plasmid was transfected into CV-1 cells infected with vΔC16ΔC4, and 2 days later rVACVs with the desired genotype were identified and plaque was purified on BS-C-1 cells as described (68, 69). For the construction of vΔC16/C4-N-TAP, an overlapping PCR was used to assemble the required plasmid. This involved generating a 5′ DNA fragment by PCR using oligonucleotides 5′-ATC GAA GCT TGC ACA CAT AAT CCA TTC TCT C-3′ (left arm, LA) and 5′-GCG GCC GCG TGG TCT CAT CCT CA-3′ and a 3′ fragment with 5′-**TGA GGA TGA GAC CAC GCG GCC GC**G TAT TGG ATT CTG CAT ATA GAT GGC-3′, containing complementary sequence to the 5′ fragment (bold underlined), and 5′-CGC GAA TTC TCC GAT CCA CCC ACG TGT CAG AT-3′ (right arm, RA) using pUC13-C4TAPRev as a template. These two fragments were joined by PCR using oligonucleotides LA and RA and cloned into pUC13-EcoGPTEGFP (69). This plasmid was used to generate the rVACV as above. The genotype of the rVACVs was analyzed by PCR following proteinase K treatment of infected BS-C-1 cells using oligonucleotides LA and RA (Fig. S4).

### Transfection of cell lines

HEK293T and HeLa cells were transfected with PEI (CellnTec, 3 µL per μg of DNA) and with TransIT-LT1 (Mirus), respectively, according to the manufacturer’s instructions.

### Reporter gene assays

HEK293T or HeLa cells were seeded in 96-well plates, and 24–48 h later, HeLa cells were transfected with 45 ng of the NF-κB promoter-luciferase reporter plasmid (NF-κB-Luc) or 150 ng of AP-1 promoter-luciferase reporter plasmid (AP-1-Luc) in HeLa cells, or HEK293T cells were transfected with 250 ng of AP-1-Luc in HEK293T cells. Each cell type was also transfected together with a TK-*Renilla* plasmid and the plasmids expressing the required VACV proteins. The exact amounts of TK-*Renilla* and expression plasmids used are included in figure legends. DMEM supplemented with 2% (vol/vol) FBS was then added to achieve a volume of 100 µL per well. Cells were incubated at 37°C for 24 h before stimulation or infection. For NF-κB activation, cells were mock-stimulated or stimulated with TNF-α at 15 ng/mL for 8 h. For AP-1 activation, cells were mock-treated or treated with phorbol 12-myristate 13-acetate (Sigma) at 10 ng/mL for 24 h. Then, the cells were washed with 200 µL of PBS per well and harvested in 100 µL per well of passive lysis buffer (Promega). Cells were freeze-thawed and 10 µL of lysate from each well was added to 50 µL of firefly luciferase substrate [20 mM Tricine, 0.1 mM EDTA, 2.67 mM MgSO_4_.7H_2_O, 33.3 mM dithiothreitol (DTT), 530 µM ATP, 5 mM NaOH, 270 µM acetyl coenzyme A, 132 µg/mL luciferin, and 0.26 mM (MgCO_3_)4Mg(OH)2.5H_2_O] or 2 µg/mL *Renilla* luciferase substrate (Prolume). Measurement of luciferase activity was carried out using a FLUOstar Omega instrument. Cells were transfected in triplicate, quadruplicate, or sextuplicate as indicated in each figure legend. Then, each firefly-luciferase reading was normalized to its corresponding *Renilla*-luciferase reading. To calculate fold inductions, these normalized values were divided by the non-stimulated empty-vector control value.

### CRISPR/Cas9-mediated genome editing

The guide RNAs (gRNAs) were designed using online software (https://portals.broadinstitute.org/gpp/public/analysis-tools/sgrna-design) to target exon 3 of both FLNB and FLNA, which is shared by all isoforms. gRNA complex for FLNB: 5′-CAC CGG GTC CCA GGA TTC CCA GTC T-3′ and 5′-AAA CAG ACT GGG AAT CCT GGG ACC C-3′. gRNA complex for FLNA: 5′-CAC CGT CCT CCT CCT CGT CCC ACA T-3′ and 5′-AAA CAT GTG GGA CGA GGA GGA GGA C-3′. CRISPR/Cas9-mediated genome editing of HeLa cells was performed as described (65). Briefly, the oligo complexes were annealed, phosphorylated, and then ligated into the PX459 CRISPR/Cas9 vector. Plasmids with or without the gRNA sequences were transfected into HeLa cells and cells were incubated for 48 h. Then, puromycin (2 µg/mL) was added for another 48 h, and puromycin-resistant cells were seeded in 96-well plates at a density of 0.5 cell/well and were left to form colonies for 2–3 weeks. Several clones were expanded, and potential knockout clones were selected by immunoblotting and confirmed by DNA sequencing at the gRNA target sites. Clones with multiple peaks in their genomic DNA sequences were cloned into the TOPO TA vector (Thermo Scientific) and sent for further sequencing (*n* = 10) to identify the alterations in both alleles. All clones selected were confirmed to have frameshift or nonsense mutations in both alleles, and no WT allele was detected. The cell lines used in this study were FLNB clone 13 (KO1), clone 17 (KO2), and FLNA clone 13.

### Immunoprecipitation and affinity purification

HEK293T or HeLa cells were seeded in 10-cm dishes and transfected with the indicated plasmids as described above. After 16–24 h, cells were washed once with PBS and then lysed with 0.5% NP-40 in PBS, IP buffer [150 mM NaCl, 20 mM Tris-HCl (pH 7.4), 10 mM CaCl_2_, 0.1% Triton-X100, and 10% [vol/vol] glycerol], or HEPES IP buffer (10 mM HEPES pH 7.4, 150 mM NaCl, and 0.5% NP-40) supplemented with cOmplete Mini EDTA-free Protease Inhibitor Cocktail (Roche). Insoluble material was removed by centrifugation at 21,000 × *g* for 20 min at 4°C and discarded. The lysates were then incubated with Anti-HA Agarose (Sigma Aldrich), ANTI-FLAG M2 Affinity Gel (Sigma Aldrich), or with streptavidin agarose gel beads (Thermo Scientific) for 16 h at 4°C. Subsequently, the beads were washed once with PBS and three times with the lysis buffer, and finally, they were incubated at 100°C for 5 min in Laemmli loading buffer to elute bound proteins. Cleared lysates and IP eluates were analyzed by SDS-PAGE and immunoblotting. Data shown are representative of at least three independent experiments showing similar results.

### SDS-PAGE and immunoblotting

Cells were lysed in 0.5% NP-40 in PBS, IP, or RIPA buffer (50 mM Tris-HCl pH 8, 150 mM NaCl, 1% NP-40, 0.5% sodium deoxycholate, and 0.1% SDS), rotated for 20 min at 4°C, and then denatured for 5 min at 100°C in the presence of Laemmli loading buffer. The lysates were then resolved by SDS-PAGE and transferred to nitrocellulose membranes (GE Healthcare) using a Trans-Blot Semi-Dry Transfer Unit (Bio-Rad). Membranes were blocked in 0.1% Tween PBS supplemented with 5% (wt/vol) skimmed milk and subjected to immunoblotting with the following primary antibodies at the indicated dilutions: Filamin 1 (FLNA) [Santa Cruz (sc-271440), mouse monoclonal; 1:200]; Filamin 3 (FLNB) [Santa Cruz (sc-271561), mouse monoclonal; 1:500]; Ku70 [Abcam (ab3114), mouse monoclonal; 1:1,000]; C16 [(17), rabbit polyclonal, 1:1,000]; FLAG [Sigma-Aldrich (F7425), rabbit polyclonal; 1:2,500]; HA [BioLegend (16B12), mouse monoclonal; 1:1,000]; α-tubulin [Merck Millipore (05-829), mouse monoclonal; 1:5,000]; β-actin [Sigma-Aldrich (A2066), rabbit polyclonal; 1:1,000]. Primary antibodies were detected using IRDye-conjugated secondary antibodies in an Odyssey Infrared Imager (LI-COR Biosciences). All secondary antibodies were used at 1:10,000 dilution.

### Tandem mass tagging quantitative proteomics

HEK293T cells in 10-cm dishes were transfected with 8.5 µg/plasmid of the TAP-tagged C4/EV, or 7 µg/plasmid of the HA-tagged FLNB/A using PEI. Sixteen hours post-transfection, the cells were infected with either v∆C4∆C16 (C4 experiment) at an MOI of 5 or vC4-TAP (FLNB/A experiment) at an MOI of 10 for 7 h. Previously, these viruses had been purified by sedimentation through a cushion of 36% (wt/vol) sucrose. Cells were washed once in ice-cold PBS, lysed in either HEPES IP buffer (10 mM HEPES pH 7.4, 150 mM NaCl, and 0.5% NP-40) (C4 experiment), or TBS IP buffer (25 mM Tris-HCl pH 7.5, 150 mM NaCl, and 0.5% NP-40) (FLNB/A experiment) supplemented with cOmplete Mini EDTA-free protease inhibitor cocktail (Roche). Lysates were then subjected to IP with either FLAG or HA beads as described above. IP samples were sent to the NERC Life Sciences Mass Spectrometry Facility at the University of Bristol where they were reduced (10 mM TCEP, 55°C for 1 h), alkylated (18.75 mM iodoacetamide, room temperature for 30 min), and then digested from the beads with trypsin (2.5 µg trypsin, 37°C, overnight). The resulting peptides were then labeled with TMT six- or 10-plex reagents according to the manufacturer’s protocol (Thermo Fisher Scientific), and the labeled samples were pooled and desalted using a SepPak cartridge according to the manufacturer’s instructions (Waters). The eluate was then fractionated using an Ultimate 3000 nanoHPLC system in line with an Orbitrap Fusion Tribrid mass spectrometer (Thermo Scientific). All spectra were acquired using Xcalibur 2.1 software (Thermo Scientific) and operated in data-dependent acquisition mode using an SPS-MS3 workflow. FTMS1 spectra were collected at a resolution of 120,000, with an automatic gain control (AGC) target of 200,000 and a max injection time of 50 ms. Precursors were filtered with an intensity threshold of 5,000, according to charge state (to include charge states 2–7), and with monoisotopic peak determination set to peptide. Previously interrogated precursors were excluded using a dynamic window (60 s, ±10 parts per million, ppm). The MS2 precursors were isolated with a quadrupole isolation window of 1.2 *m/z*. ITMS2 spectra were collected with an AGC target of 10,000, max injection time of 70 ms, and collision-induced dissociation collision energy of 35%. For FTMS3 analysis, the Orbitrap was operated at 50,000 resolution with an AGC target of 50,000 and a max injection time of 105 ms. Precursors were fragmented by high energy collision dissociation at a normalized collision energy of 60% to ensure maximal TMT reporter ion yield. Synchronous Precursor Selection was enabled to include up to 5 MS2 fragment ions in the FTMS3 scan.

### Mass spectrometry analysis

To analyze the mass spectrometry data for the C4 experiment, raw files were processed and quantified using Maxquant v1.6.14 (70) and searched against the UniProt Human database (downloaded September 2016: 70,550 entries) plus the entire VACV strain WR proteome sequence (NCBI accession NC_006998.1). Precursor mass tolerance was set at 4.5 ppm, and MS/MS tolerance was set at 0.5 Da. For the analysis of FLNB/A experiment, raw data files were processed and quantified using the Proteome Discoverer software v2.1 (Thermo Scientific) and searched against the UniProt Human database (downloaded September 2018: 152,927 entries) and entire VACV strain WR proteome sequence (NCBI accession NC_006998.1) using the SEQUEST HT algorithm. Peptide precursor mass tolerance was set at 10 ppm, and MS/MS tolerance was set at 0.6 Da. For both experiments, search criteria included oxidation of methionine (+15.995 Da), acetylation of the protein N terminus (+42.011 Da), and methionine loss plus acetylation of the protein N terminus (−89.03 Da) as variable modifications. As fixed modifications, carbamidomethylation of cysteine (+57.021 Da) and the addition of the TMT mass tag (+229.163 Da) to peptide N termini and lysine were included. Searches were performed with full tryptic digestion and a maximum of two missed cleavages were allowed. The reverse database search option was enabled, and all data were filtered to satisfy a false discovery rate of 5%. Contaminants, reverse database hits, and hits corresponding to a single peptide were removed. Entries that did not have an identified abundance in any of the samples were deleted. Downstream analysis was carried out in the Perseus software (71). Label intensities were converted to Log2 values, which were then used to perform a median normalization. To account for missing data, an imputation took place from the normal distribution. Finally, samples were compared and Log2 values were subtracted to calculate the fold change.

### Protein expression and purification

All proteins were expressed in B834(DE3)pLysS *E. coli* cells (Novagen). Bacteria were grown at 37°C in a 2× TY medium with shaking at 200 rpm to an OD_600_ of 0.7–0.9. FLNB expression was induced by adding 0.5 mM IPTG and incubating at 37°C for 4 h. C4-N expression was induced by cooling the cultures to 22°C, adding 0.5 mM IPTG, and incubating overnight. Cells were harvested by centrifugation at 5,000 × *g* for 15 min, and the cell pellets were stored at −80°C. Cells were thawed and resuspended in lysis buffers containing either 20 mM HEPES pH 7.0 (FLNB) or 20 mM Tris pH 9.0 (C4-N) and 500 mM NaCl, 1 mM β-mercaptoethanol, 0.05% Tween-20, 0.5 mM MgCl_2_, 400 U bovine DNase I (Roche), and one tablet of complete EDTA-free protease inhibitor (Sigma-Aldrich). Cells were lysed by passage through an Avestin EmulsiFlex-C5 Emulsifier (ATA Scientific) at 80 psi three to four times. Lysates were collected and cleared by centrifugation at 40,000 × *g* for 30 min at 4°C. Cleared lysates were applied to a 5 mL HiTrap TALON crude column (GE Healthcare) pre-equilibrated with binding buffer containing either 20 mM HEPES pH 7.0 for FLNB or Tris pH 9.0 for C4-N and 500 mM NaCl, 10 mM imidazole, and 1 mM DTT to capture the His6-tagged proteins. The columns were washed sequentially with binding buffers containing 25 and 50 mM imidazole, respectively, to remove non-specifically bound contaminants. The bound proteins were eluted by washing the column with binding buffers containing 500 mM imidazole. Eluents were concentrated and further purified by SEC using a Superdex 200 (for FLNB) or Superdex 75 (for C4-N) column (GE Healthcare) equilibrated in gel filtration buffer (20 mM HEPES pH 7.0, 1 mM DTT, and 200 mM NaCl for FLNB or 500 mM NaCl for C4-N).

### Analytical size-exclusion chromatography

Analytical SEC experiments were performed at room temperature. For each experiment, 100 µL protein at 1 mg/mL was injected onto a Superdex 200 increase 10/300 GL column (GE Healthcare) pre-equilibrated with 20 mM HEPES pH 7.0, 200 mM NaCl, and 1 mM DTT at a flow rate of 0.5 mL/min. The column was calibrated with protein standards blue dextran, β-amylase, alcohol dehydrogenase, albumin, carbonic anhydrase, and cytochrome C (all from Sigma). The retention volume for each elution peak was recorded.

### Isothermal titration calorimetry

ITC studies were performed at 25°C on an automated MicroCal PEAQ-ITC (Malvern Panalytical). Proteins were buffer-exchanged into 20 mM HEPES pH 7.0, 200 mM NaCl, and 1 mM DTT by SEC. FLNB R22-24 and R23-24 (titrants) at concentrations of 80, 100, and 130 µM were titrated into 8, 10, and 13 µM of C4-N 1–149 (titrate) in a series of 13 × 3, 19 × 2, or 25 × 1.5 µL injections, respectively. The results were analyzed using the MicroCal PEAQ-ITC analysis software (Malvern Panalytical), and a one-site binding model was used to fit the data.

### Analysis of plaque size

Monolayers of WT or FLNA^−/−^ or FLNB^−/−^ HeLa cells or MEFs in 6-well plates were infected with VACV A5-GFP to give well-separated plaques. The infected cells were overlaid with a 50:50 mix of MEM and 2% (wt/vol) CMC, and 2 days later, GFP plaques were imaged on a Zeiss Axiovert 200 M microscope (Zeiss), and the area of plaques was measured using ImageJ software.

### Analysis of virus growth kinetics

Monolayers of FLNB WT or FLNB^−/−^ HeLa cells in 6-well plates were infected at either MOI 5 or 0.01 with vC4-TAP. After 1 h at 37°C, inocula were removed and cells were either scraped into fresh medium (1 h time point), or extra medium was added and after 24 or 48 h, cells were scraped in their medium and kept at −70°C. For the grown kinetics in MEFs, monolayers of WT FLNB, WT FLNA, FLNA^−/−^, or FLNB^−/−^ MEFs in T25 flasks were infected at an MOI of 0.01 with vC4-TAP. After 1.5 h at 37°C, cells were washed and either scraped into their medium and collected by centrifugation at 500 × *g* for 10 min or were incubated for 24 or 48 h, after which the medium was removed, and cells were collected by centrifugation. All samples were frozen and thawed three times and sonicated, and the infectious virus titer was determined by plaque assay on BS-C-1 cells.

### Murine intranasal infection

All viruses used for *in vivo* work were purified by sequential sedimentation through two sucrose cushions (36% wt/vol) prior to inoculation. Groups of BALB/c mice 6–8 weeks old (*n* = 5) were anesthetized and inoculated with 1 × 10^5^ p.f.u. into both nostrils. Weight loss was measured daily up to 12 days pi.

### Cell-to-cell distances

WT and FLNB^−/−^ HeLa cells were seeded onto 10-mm coverslips and incubated for 24 h. The growth medium was then aspirated, and cells were washed three times with cold PBS. Cells were fixed with 4% (wt/vol) paraformaldehyde (PFA) in 250 mM HEPES (pH 7.4) for 15 min on ice, followed by 8% (wt/vol) PFA for 30 min at RT. Fixed cells were blocked with 10% FBS in PBS and permeabilized with 0.1% Triton X-100. Finally, the cells were mounted [10% (wt/vol) Mowiol 4-88 (CalBiochem), 25% (vol/vol) glycerol, 100 mM Tris-HCl pH 8.5, and 0.5 µg/mL DAPI (Sigma)] and imaged using a Zeiss LSM780 confocal laser scanning microscopy system mounted on an AxioObserver.Z1 inverted microscope using a ×60 objective and Zen acquisition software. The distances between nuclei of neighboring cells (*n* = 19 per cell line) were measured.

### Statistical analysis

Statistical significance was determined using an unpaired Student’s *t*-test with Welch’s correction where appropriate, by using GraphPad Prism 7 software. Experiments with cultured cells were carried out at least twice, and the *in vivo* experiments were performed twice. One representative data set of each experiment is shown, and all error bars represent the standard error of the mean.

## Data Availability

Data for this submission have been deposited at Figshare, DOI: https://doi.org/10.6084/m9.figshare.22927847.v1. The mass spectrometry proteomics data have been deposited to the ProteomeXchange Consortium (http://proteomecentral.proteomexchange.org) via the iProX partner repository (72, 73) with the data set identifier PXD042378.

## SUPPLEMENTAL MATERIAL

Fig. S1 jvi.01485-23-s0001.pdf Relative FLNA and FLNB abundance during VACV infection.

Fig. S2 jvi.01485-23-s0002.pdf TMT data for FLNA and FLNB during VACV infection.

Fig. S3 jvi.01485-23-s0003.pdf C4 does not bind R22-R23 or R23.

Fig. S4 jvi.01485-23-s0004.pdf PCR analysis of VACV genomes.

Fig. S5 jvi.01485-23-s0005.pdf Plaques in FLNB WT and FLNB-/- cell lines.

Fig. S6 jvi.01485-23-s0006.pdf Cell-to-cell distance of FLNB WT and FLNB-/- cells.

Fig. S7 jvi.01485-23-s0007.pdf Plaques in FLNA WT and KO cell lines.

ASM does not own the copyrights to Supplemental Material that may be linked to, or accessed through, an article. The authors have granted ASM a non-exclusive, world-wide license to publish the Supplemental Material files. Please contact the corresponding author directly for reuse.
