# Supplementary material for: Filamin B restricts vaccinia virus spread and is targeted by vaccinia virus protein C4
Source: J Virol. 2024 Feb 27;98(3):e01485-23. doi: 10.1128/jvi.01485-23 (PMC10949515; doi:10.1128/jvi.01485-23)
Supplement: Fig. S1 — Relative FLNA and FLNB abundance during VACV infection. [file jvi.01485-23-s0001.pdf]

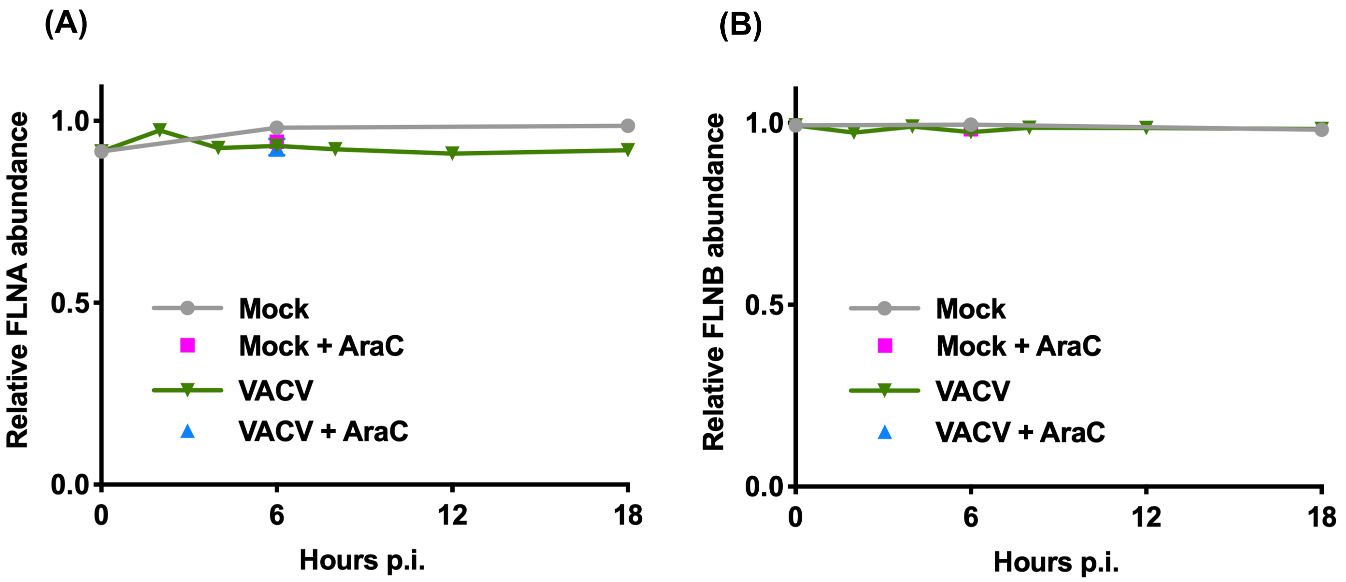

Fig. S1: Relative FLNA and FLNB abundance during VACV infection.

Figs were generated by using data from Table S1 from Soday et al., 2019 [36]. Briefly, cells HFFF-TERT cells were infected in triplicate at MOI 5 with VACV WR, or mock-infected, and samples were collected at the indicated times pi. In addition, one mock and one infected sample were treated with the cytosine arabinoside (AraC) for 6 h. The relative abundance of FLNA **(A)** or FLNB **(B)** from all three replicates is shown as mean.
