# Supplementary material for: Filamin B restricts vaccinia virus spread and is targeted by vaccinia virus protein C4
Source: J Virol. 2024 Feb 27;98(3):e01485-23. doi: 10.1128/jvi.01485-23 (PMC10949515; doi:10.1128/jvi.01485-23)
Supplement: Fig. S2 — TMT data for FLNA and FLNB during VACV infection. [file jvi.01485-23-s0002.pdf]

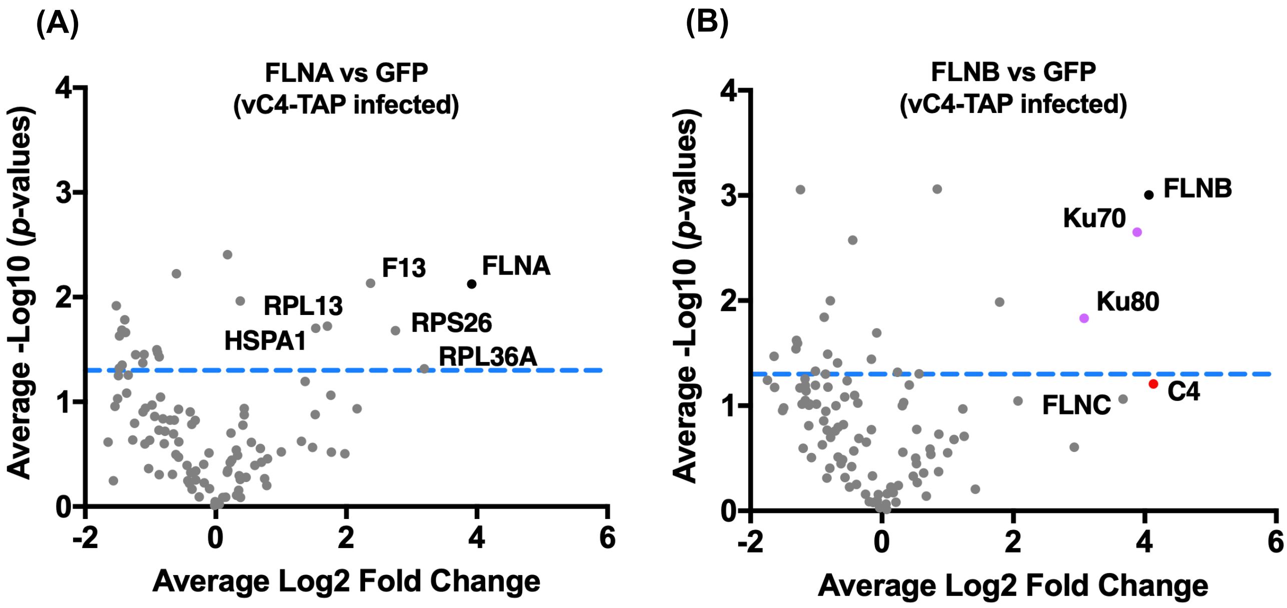

Fig. S2: TMT data for FLNA and FLNB during VACV infection.

HEK293T cells were transfected in triplicate with HA-tagged FLNA, FLNB or GFP for 16 h and then cells were infected at MOI 10 with vC4-TAP for 7 h. Cells were then lysed and subjected to HA IP. Log2 ratios of the mean of 3 HA-FLNA **(A)** or HA-FLNB **(B)** pulldowns against HA-GFP pulldown (x-axis) are plotted versus −Log10 of the *p*-values (y-axis) derived from a Student's *t*-test. The threshold of *p*-value=0.05 is indicated with the blue dashed line. FLNA and FLNB baits are shown with black dots, C4 hit is indicated in red, and C4's partners Ku70/80 are in purple.
