# Supplementary material for: Filamin B restricts vaccinia virus spread and is targeted by vaccinia virus protein C4
Source: J Virol. 2024 Feb 27;98(3):e01485-23. doi: 10.1128/jvi.01485-23 (PMC10949515; doi:10.1128/jvi.01485-23)
Supplement: Fig. S3 — C4 does not bind R22-R23 or R23. [file jvi.01485-23-s0003.pdf]

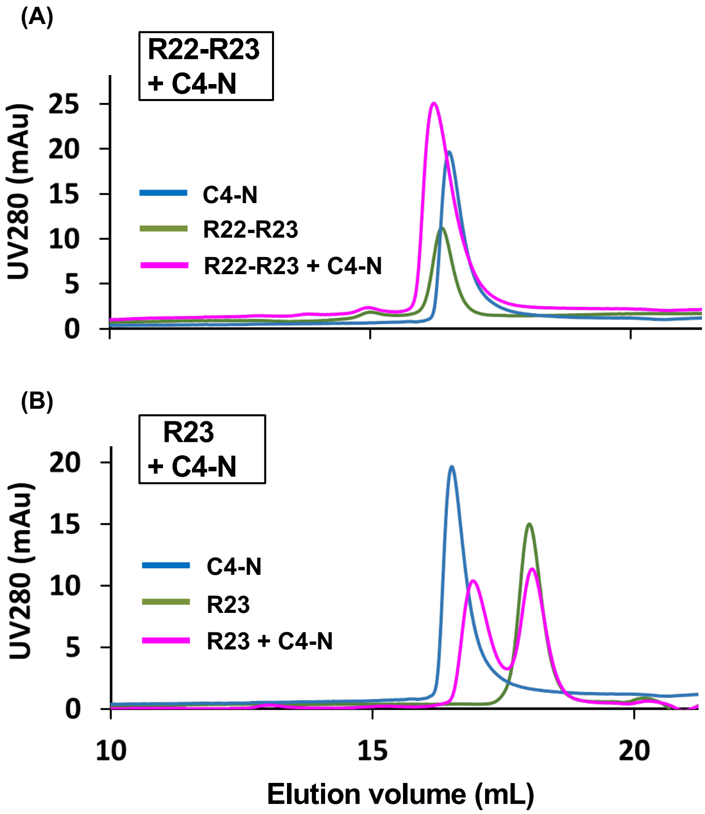

Fig. S3: C4 does not bind R22-R23 or R23.

(A) SEC chromatograms showing elution profiles of R22-R23 (green trace), C4-N (blue trace) and R22-R23 + C4-N mixture (at 1:1 molar ratio, pink trace) from a Superdex 200 Increase 10/300 GL column. **(B)** SEC chromatograms showing elution profiles of R23 (green trace), C4-N (blue trace) and R23 + C4-N mixture (at 1:1 molar ratio, pink trace) from a Superdex 200 Increase 10/300 GL column.
