# Supplementary material for: Filamin B restricts vaccinia virus spread and is targeted by vaccinia virus protein C4
Source: J Virol. 2024 Feb 27;98(3):e01485-23. doi: 10.1128/jvi.01485-23 (PMC10949515; doi:10.1128/jvi.01485-23)
Supplement: Fig. S4 — PCR analysis of VACV genomes. [file jvi.01485-23-s0004.pdf]

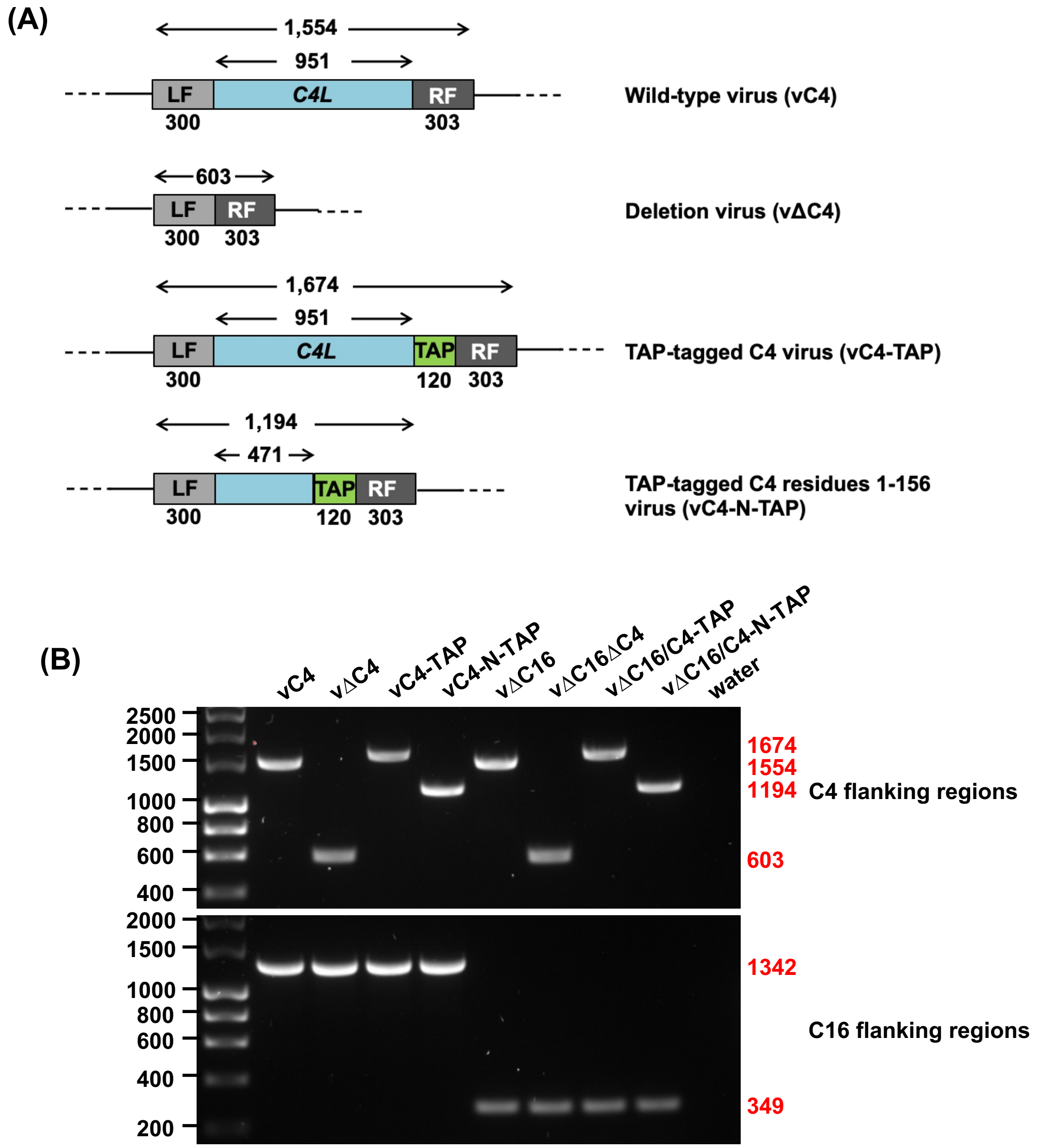

Fig. S4: PCR analysis of VACV genomes.

(A) Schematic representation of the VACV strain WR *C4L* locus. Expected sizes of PCR products are indicated in bp between outward pointing pairs of arrows. Grey boxes represent the left and right flanking regions. Blue and green boxes represent the *C4L* ORF and the sequence encoding the TAP tag, respectively. **(B)** BS-C-1 cells were infected with the indicated viruses for 24 h and then cells were scraped into a solution containing proteinase K. Genomic DNA was extracted from virion cores by proteinase K digestion and this was used as a template for PCR with *C4L* flanking primers (top panel) and *C16L* primers (lower panel). PCR products were resolved by agarose gel electrophoresis. The positions of DNA size markers are shown on the left and expected PCR sizes (in bps) on the right (red).
