# Supplementary material for: Filamin B restricts vaccinia virus spread and is targeted by vaccinia virus protein C4
Source: J Virol. 2024 Feb 27;98(3):e01485-23. doi: 10.1128/jvi.01485-23 (PMC10949515; doi:10.1128/jvi.01485-23)
Supplement: Fig. S5 — Plaques in FLNB WT and FLNB-/- cell lines. [file jvi.01485-23-s0005.pdf]

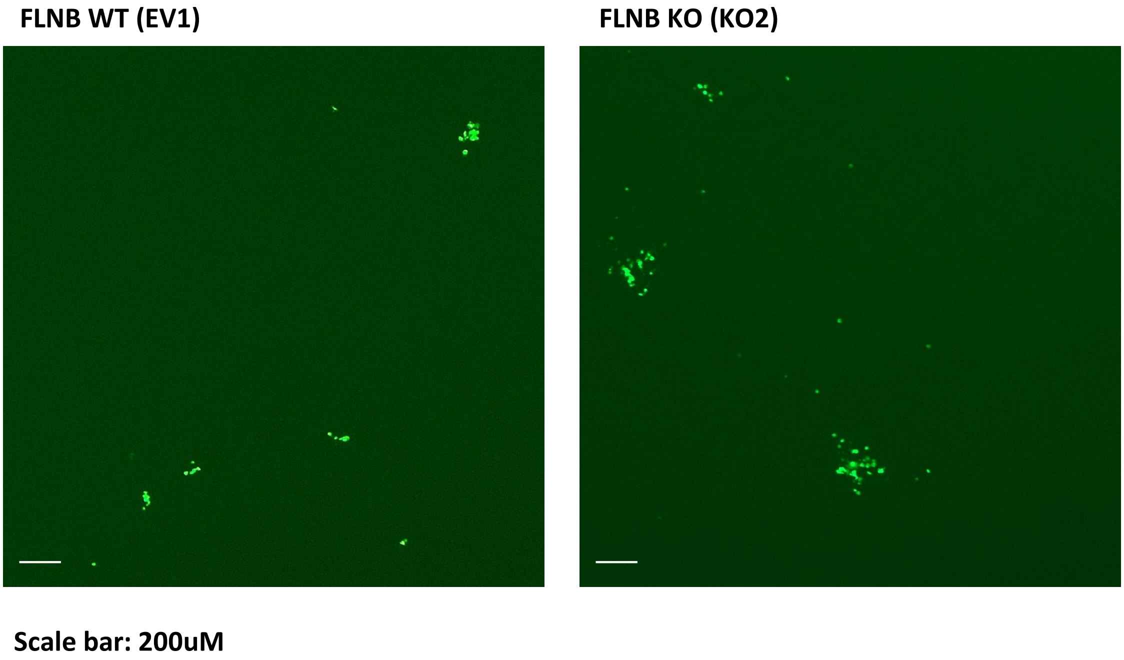

Fig. S5: Plaques in FLNB WT and KO cell lines.

Monolayers of HeLa WT or FLNB$^{-/-}$ cells were infected with VACV A5-GFP to give distinct plaques. Images of GFP-expressing plaques were imaged 2 d later on a Zeiss Axiovert 200 M microscope (Zeiss) and representative images are shown.
