# Supplementary material for: Filamin B restricts vaccinia virus spread and is targeted by vaccinia virus protein C4
Source: J Virol. 2024 Feb 27;98(3):e01485-23. doi: 10.1128/jvi.01485-23 (PMC10949515; doi:10.1128/jvi.01485-23)
Supplement: Fig. S6 — Cell-to-cell distance of FLNB WT and FLNB-/- cells. [file jvi.01485-23-s0006.pdf]

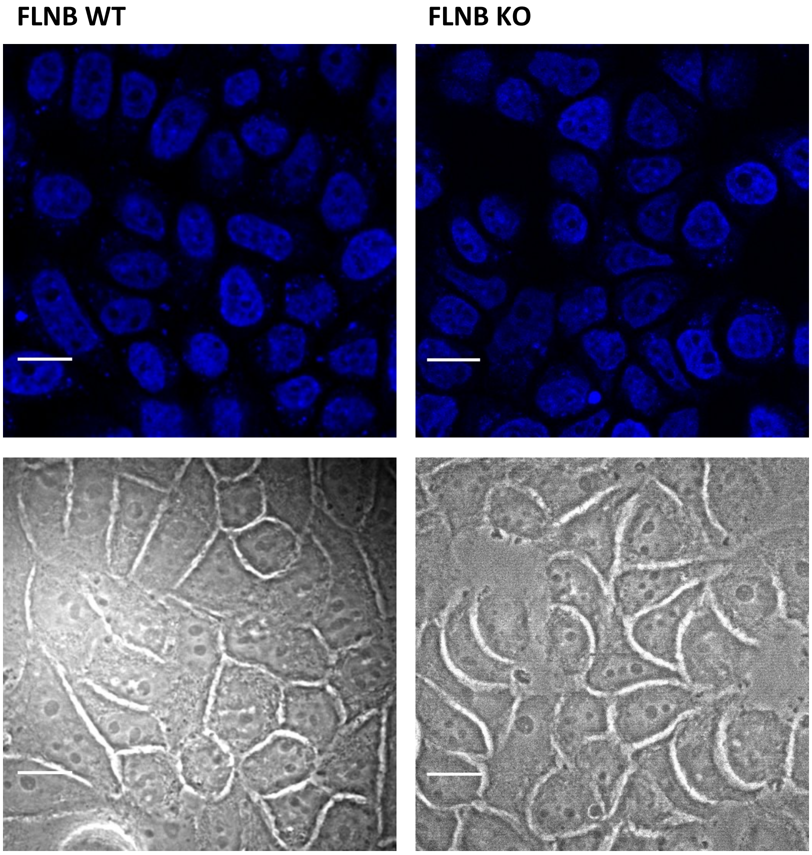

Scale bar: 15uM

Fig. S6: Cell-to-cell distance of FLNB WT and KO cells.

Nuclei distances of HeLa WT or $FLNB^{-/-}$ cells were measured in confocal microscopy, as a way to assess cell-to-cell distance. DAPI (top panels) and brightfield (bottom panels) are shown.
