# Supplementary material for: Filamin B restricts vaccinia virus spread and is targeted by vaccinia virus protein C4
Source: J Virol. 2024 Feb 27;98(3):e01485-23. doi: 10.1128/jvi.01485-23 (PMC10949515; doi:10.1128/jvi.01485-23)
Supplement: Fig. S7 — Plaques in FLNA WT and KO cell lines. [file jvi.01485-23-s0007.pdf]

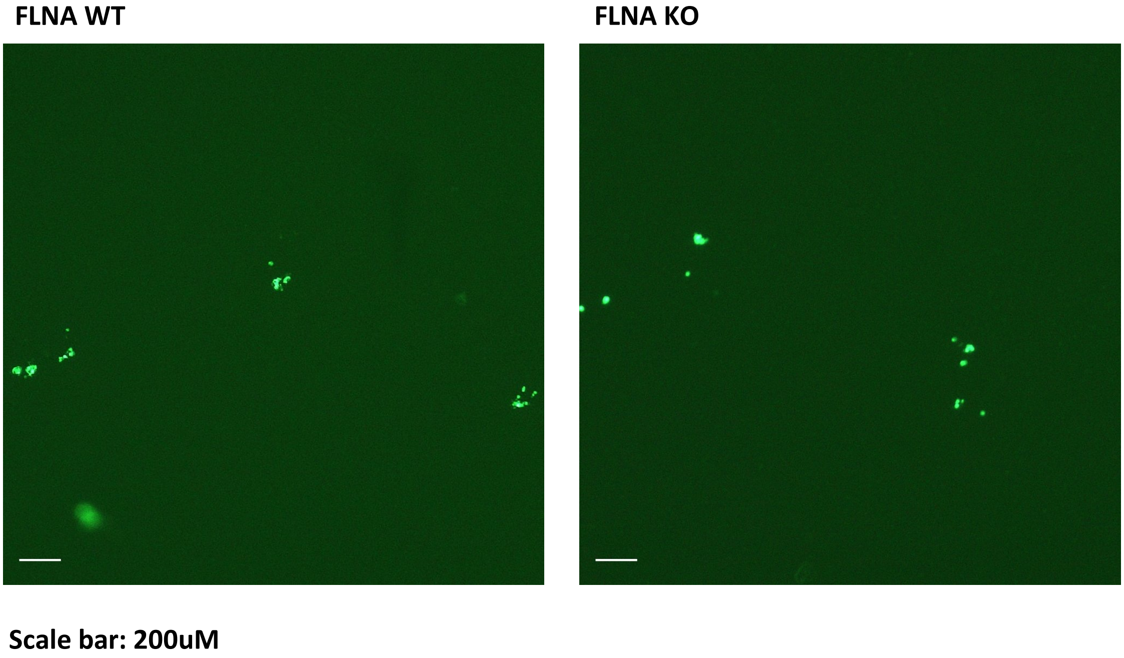

Fig. S7: Plaques in FLNA WT and KO cell lines.

HeLa WT or FLNA$^{-/-}$ cells were infected with VACV A5-GFP and plaques were corded 2 d later. Images of GFP-expressing plaques were imaged on a Zeiss Axiovert 200 M microscope (Zeiss) and representative images are shown.
